# Oxidative Stress-Mediated Overexpression of Uracil DNA Glycosylase in *Leishmania donovani* Confers Tolerance against Antileishmanial Drugs

**DOI:** 10.1155/2018/4074357

**Published:** 2018-02-25

**Authors:** Anshul Mishra, Mohd. Imran Khan, Pravin K. Jha, Ajay Kumar, Sushmita Das, Prolay Das, Pradeep Das, Kislay K. Sinha

**Affiliations:** ^1^Department of Biotechnology, National Institute of Pharmaceutical Education and Research, Hajipur 844102, India; ^2^Rajendra Memorial Research Institute of Medical Sciences (RMRIMS), Agamkuan, Patna, Bihar 800007, India; ^3^Department of Microbiology, All India Institute Medical Sciences (AIIMS), Patna, Bihar 801507, India; ^4^Department of Chemistry, Indian Institute of Technology (IIT), Patna, Bihar 801106, India

## Abstract

*Leishmania donovani* is an intracellular protozoan parasite that causes endemic tropical disease visceral leishmaniasis (VL). Present drugs used against this fatal disease are facing resistance and toxicity issues. Survival of leishmania inside the host cells depends on the parasite's capacity to cope up with highly oxidative environment. Base excision repair (BER) pathway in *L. donovani* remains unexplored. We studied uracil DNA glycosylase (UNG), the key enzyme involved in BER pathway, and found that the glycosylase activity of recombinant LdUNG (*Leishmania donovani* UNG) expressed in *E. coli* is in sync with the activity of the parasite lysate under different reaction conditions. Overexpression of UNG in the parasite enhances its tolerance towards various agents which produce reactive oxygen species (ROS) and shows a higher infectivity in macrophages. Surprisingly, exposure of parasite to amphotericin B and sodium antimony gluconate upregulates the expression of UNG. Further, we found that the drug resistant parasites isolated from VL patients show higher expression of UNG. Mechanisms of action of some currently used drugs include accumulation of ROS. Our findings strongly suggest that targeting LdUNG would be an attractive therapeutic strategy as well as potential measure to tackle the problem of drug resistance in the treatment of leishmaniasis.

## 1. Introduction

Leishmaniasis is an intricate parasitic infection instigated by more than 20 different species of protozoa belonging to genus *Leishmania* [1]. Worldwide, 350 million people are at risk with 3 different major forms of leishmaniasis: visceral leishmaniasis (VL), cutaneous, and mucosal. Annual incidence of VL is estimated 50,000 to 90,000 new cases, majority of which is reported from six countries: India, Sudan, South Sudan, Bangladesh, Brazil, and Ethiopia [2–4].

Pentavalent antimonials, especially sodium stibogluconate (SSG), had been the choice of drug against VL; however, due to severe side effects and emergence of resistance, standard treatment was shifted to amphotericin B (AmpB). AmpB, a second line drug having a higher cure rate, is accompanied by adverse effects such as high fever, nausea, vomiting, and nephrotoxicity [5]. Nevertheless, significant progress has been made during the past two decades; in addition to AmpB, deoxycholate, liposomal AmpB, miltefosine, and paromomycin have been developed and registered in various countries for the treatment of VL [6]. Studies are also underway to test combinations of various antileishmanial drugs to maintain high efficacy, delay the development of drug resistance, shorten treatment duration, and reduce toxicity and cost. Inspite of all these, inventory of antileishmanial drugs is very small, and common relapses, long treatment course (miltefosine, 28 days; paromomycin, 21 days), and prolonged secondary prophylaxis regimens could favour the emergence of resistant strains. These limit therapeutic options even further and create an epidemiological issue in the area of anthroponotic VL transmission [6, 7]. Limitations of currently used drugs are parenteral administration (except miltefosine), toxicity, long course of treatment, resistance, need for hospitalization, close monitoring, cost, and emergence of HIV/VL coinfection [8]. All these factors together make the search for new drug targets very vital to cure leishmaniasis.

Transmission vector of leishmania parasite is Phlebotomus sand fly. The parasite completes its digenetic life cycle with two morphological forms: the slender flagellated promastigote and the oval intracellular amastigote [9]. Infected sand flies introduce the promastigote stage, that is, metacyclic form of the parasite, into healthy human through bloodstream of the vertebrate host when they bite them to take a blood meal. After entering into an alien environment, the flagellated parasite invades the host macrophages for initial survival and then gets transformed into a nonmotile amastigote stage, with subsequent spread of parasite to neighbouring macrophages. The host offers hostile oxidative environment to the parasites which in turn have different survival strategies inside the host macrophages, like retreat into a safe intracellular compartment [10, 11], modulation of macrophage apoptosis by enhancing its survival [12], inhibition of antigen presentation, and T-cell stimulation by reduced expression of MHC class II and costimulatory molecules by macrophages [13–15]. The host-infected macrophages produce reactive oxygen species (ROS) and iNOS (cytokine inducible nitric oxide synthases) which pose enormous continuous threat to the genomic integrity of parasite. To escape this detrimental effect, parasite necessitates super scrutiny system and this leads to existence of a robust frame containing various DNA repair pathways to ensure its survival. DNA repair is a group of numerous multienzyme, multistep processes that is required to protect the cellular genomic integrity from genotoxic insults. Majority of lesions produced in this oxidative environment includes modified bases, misincorporated uracil, abasic sites, and single-strand breaks and various aspects of these damages and repair have been recently reviewed [16]. Oxidatively generated damage and dUTP misincorporation are important sources of mutation in living cells. Most of the endogenous deleterious lesions are repaired mainly by BER system. It is most versatile among excision repair pathways, and it is conserved across the species. Overall, most of the features and enzymatic reactions are similar for BER pathway, yet there are subtle but key differences observed from organism to organism. As reported in other eukaryotes, BER involves 4-5 steps starting with base excision by a DNA glycosylase, followed by a common pathway usually involving an AP-endonuclease (APE) to generate 3′ OH terminus at the damage site, followed by repair synthesis with a DNA polymerase and nick sealing by a DNA ligase [17, 18].

*Leishmania sp*. is sensitive to oxidative stress, in part because they lack the enzymes, catalase, and glutathione peroxidase, in their promastigote form [19]. This sensitivity makes leishmania BER system a potential target for antiparasitic drugs. However, little is known about the contribution of the BER pathway in life cycle of this parasite. Studies are required to establish the role of genes involved in BER pathway in cell viability, infectivity, survival, and pathogenicity of the parasite that diverged from the main line of eukaryotes at least 500 million years ago. The rate of BER of uracil is controlled by glycosylase activity of UNG in human cell lines [20].

This study aimed at deciphering the roles of UNG, a key enzyme of BER pathway in *Leishmania donovani*. The repair enzyme was characterized for its activity in different conditions of pH, temperature, salt, and cofactors. Besides oxidative stress being a major challenge presented by the host macrophage, most commonly used drugs also target the parasite by generating lethal ROS response. We studied the effects of overexpression of UNG gene on survival of parasite under various stress conditions including H_2_O_2_, UV, and menadione and compared it with those of wild-type parasite. Effect of overexpression of UNG on infectivity of the parasite was also evaluated. Expression levels of UNG in wild-type, drug-treated parasite and drug-resistant strain were also investigated to understand the role of this particular gene in development of drug resistance. Our findings suggest that UNG could be a potential target for novel drugs against leishmaniasis.

## 2. Materials and Methods

### 2.1. Animal Ethical Statement

For infectivity assessment, 6–8-week-old female BALB/c mice were used after the prior approval of the Institutional Animal Ethical Committee (IAEC) (INT-10/29-12-2015), Rajendra Memorial Research Institute of Medical Sciences (RMRIMS), Patna; Indian Council of Medical Research, New Delhi, regulated by CPCSEA; and the Government of India, New Delhi. The RMRIMS, ICMR, follows “The Guide for the Care and Use of Laboratory Animals,” 8th edition by the Institute for Laboratory Animal Research.

### 2.2. Clinical Isolates and Parasite Culture

Standard AmpB-sensitive *L. donovani* strain Ag83 (MHOM/IN/1983/AG83) was used. The promastigotes were grown at 25°C in Medium-199 media (Gibco, Carlsbad, CA, USA) supplemented with 10% foetal bovine serum (FBS) (Gibco). The culture was initiated at 1 × 10^5^ parasites/ml and grown at 24 ± 1°C in BOD incubator for 4-5 days before subculturing (late log phase). Drug-resistant clinical isolates of *L. donovani* were obtained from the splenic aspirates of VL patients unresponsive to AmpB and sodium antimony gluconate (SAG) treatments, in the indoor ward facility of RMRIMS, Patna, Bihar, India. The sensitive strains were finally maintained in M-199 medium supplemented with 10% FBS, 100 units/ml penicillin, and 100 *μ*g/ml streptomycin.

### 2.3. Preparation of Partially Purified Native LdUNG

The *L. donovani* Ag83 parasites were harvested by centrifugation at 2000*g* at 25°C for 10 min. The pellet was washed twice with phosphate-buffered saline (PBS) and kept at −80°C until used. The extraction buffer containing 50 mM Tris-HCl pH 7.6, 1 mM EDTA, 2 mM DTT, 1 mM PMSF, and 0.01% NP-40 was used to dissolve the parasite pellet in approximately 3 : 1 ratio buffer to pellet. Suspended cells were disrupted by Dounce homogenization and subsequently stirred on ice for 30 min with 0.5 M KCl to extract nucleoprotein. After centrifugation at 17,600*g* for 40 min at 4°C, the supernatant was dialyzed at 4°C for 8–10 hours against buffer A containing 25 mM Tris-HCl pH 9.0, 1 mM EDTA, 2 mM DTT, 1 mM PMSF, 0.01% NP-40, 5% sucrose, and 20% glycerol [9].

### 2.4. Cloning, Expression, and Purification of Recombinant LdUNG

The gene encoding UNG was amplified using genomic DNA as template with initial denaturing at 95°C for 5 min followed by 35 cycles of reaction with denaturing at 95°C for 1 min, annealing at 60°C for 30 seconds, extension at 72°C for 1 : 30 min after each cycle, and final extension at 72°C for 3 min. The sequences of PCR primers containing restriction sites EcoRI and HindIII are as follows: LdUNG-forward 5′ TTTTGAATTCATGCAGAAAACGCTCTTCG 3′ and LdUNG-reverse 5′ TTTTAAGCTTTCAGGGTAGCGAAGCGTTC 3′. The PCR product was analysed on 1% agarose gel. Amplified gene was cloned into pET28a into *E. coli* DH5*α* and named as pET28a-LdUNG. It was transformed into *E. coli* BL-21 (DE3) and selected on 30 *μ*g/ml kanamycin. Exponentially growing cells were induced with 0.4 mM IPTG for 4 hours at 37°C with shaking. The cells were harvested and boiled with 1X SDS-PAGE loading buffer and analysed on 10% polyacrylamide gel. The recombinant enzyme tagged with 6 × His was purified using Ni-NTA column.

### 2.5. Enzymatic Assay for LdUNG Activity

Two 47-mer complementary single-stranded oligonucleotides were synthesized containing a single uracil each at different positions (Table S1). These oligonucleotides were used as either single- or double-strand substrate for the glycosylase activity of UNG. Double-stranded substrate was prepared by annealing equimolar concentrations of 47-mer single-stranded Oligo-U (23rd position) and complementary 47-mer single-stranded Oligo-U (21st position) in 50 *μ*l reaction volumes containing 70 mM Tris-HCl pH 7.6, 10 mM MgCl_2_, and 5 mM DTT. The mixtures were heated at 95°C for 5°min and allowed to slowly cool to room temperature. The annealed substrate was purified using PCR purification kit (QIAGEN, Hilden, Germany).

The UNG activity was assayed using standard buffer (20 mM Tris-HCl pH 8.2, 1 mM EDTA, 10 mM NaCl, 2.27 nmol Oligo-U, and 50 ng of LdUNG at 37°C for 60 min). The glycosylase activity was stopped by heating the reaction mixture at 65°C for 20 min, the resultant apurinic/apyrimidinic (AP) sites were incised by alkaline treatment with 0.5 M NaOH at 37°C for 20 min, and the reaction was neutralized with 0.5 M HCl. The AP lyase activity of LdUNG was also checked through the same procedure except for hot alkaline treatment. Reaction products were run on 20% PAGE containing 7 M urea and analysed after staining with SYBR GOLD dye. The intensities of sliced substrate and product bands from the gel were measured in SYNGENE Tool software of Chemidoc (G-BOX). One unit of activity *E. coli* UDG is defined as the amount of enzyme required for releasing 1 nmol of uracil from uracil-containing DNA template in 60 min at 37°C in standard reaction buffer. The same definition was adopted for LdUNG.

Biochemical properties of recombinant LdUNG and leishmania lysate were studied based on glycosylase activity under different reaction conditions, namely, temperature, pH, salt concentration, and cofactors. The effect of temperature ranging from 4–65°C on enzyme activity was checked. LdUNG activity was also checked at different pH conditions using different buffer conditions (phosphate-citrate buffer for pH 4–7, 20 mM Tris-HCl for pH 7.5–8.5, and glycine-NaOH buffer for pH 9–10.5). Effects of salt (NaCl over the range of 5–500 mM), varying concentrations of divalent cation cofactors including MgCl_2_, MnCl_2_, and CaCl_2_ (1, 10, 50, and 100 mM), on LdUNG were investigated. Relative activities of LdUNG were observed following same protocol as above by division of intensities of product band to that of both total substrate and product. This helps to determine steady state kinetic parameter (Km) for different substrates of the recombinant LdUNG, and concentrations of substrates were varied from 0.01 to 3.5 *μ*M. The reaction was initiated by adding 20 nM LdUNG (10 ng) to the reaction mixture and incubated at 37°C for 30 min. The Michaelis-Menten equation with nonlinear least square algorithm in Sigma Plot 12.0 was used to calculate kinetic parameter (Km).

### 2.6. Construction of Expression Vectors and Transfection in *L. donovani*

The LdUNG ORF was also cloned into pLpneo2, which is a suitable expression vector for leishmania, and it was transfected into sensitive laboratory strain (Ag83) by electroporation at 25 *μ*F, 1500 V (3.75 kV/cm) with a gap of 10 seconds between pulses using Gene Pulser X-Cell (Bio-Rad) [21]. Parasites were transfected with pLpneo2 alone for control. Transfectants were selected and maintained in 100 *μ*g/ml G418. Overexpression of LdUNG in the sensitive strain was analysed by reverse transcriptase PCR (RT-PCR) with SYBR green probe.

### 2.7. Viability of Leishmania upon Exposure to Different DNA-Damaging Agents by MTT Assay

Approximately equal number (~1 × 10^7^) of late log-phase wild-type and transfected cells was treated with increasing concentrations of H_2_O_2_, menadione, UV, and sodium bisulfite, and cell viability was measured as described [22]. The UV radiation used in this study has a wavelength of 302 nm which falls under the UVB range (mainly UVA and UVB radiations are responsible for ROS generation). After treatment, the cells were pelleted at 4500*g* for 10 min and washed twice with 1X PBS. The pellets were resuspended in fresh media and incubated in BOD incubator. Inhibitory effect of oxidant treatment on parasite viability was checked by methylthiazol tetrazolium (MTT) assay, and OD of formazan was measured at 570 nm. The OD_570_ represented the cell survival, and the percentage of cell survival at each compound concentration was determined by comparing with the OD_570_ of untreated control. All experiments using transfected cells were carried out in the presence of 0.1 mg/ml G418 unless otherwise specified.

### 2.8. Quantification of AP Sites by ARP Assay

Approximately 1 × 10^7^ log-phase cells of wild-type and transfected parasites were exposed to H_2_O_2_ (200 *μ*M), menadione (20 *μ* M), and UVB (28 mJ/cm^2^). After exposure to these agents for 30 min, the cells were pelleted at 4500*g* for 10 min and washed twice with 1X PBS. The pelleted cells were again resuspended in fresh media and incubated in BOD incubator for 4 hours. The genomic DNA was isolated using QIAGEN Genomic DNA isolation kit following the manufacturer's protocol. The AP site was quantified in 1 *μ*g DNA using aldehyde reactive probe-based Oxiselect™ Oxidative DNA damage Quantitation Kit (Cell Biolabs Inc.) according to the manufacturer's instructions. The numbers of AP sites per 10^5^ nucleotides were calculated based on the linear calibration curve produced using ARP-DNA standard solutions supplied by the manufacturer.

### 2.9. RNA Extraction, Reverse Transcription, and RT-PCR

The freshly grown parasites were treated with H_2_O_2_ (200 *μ*M), menadione (20 *μ*M), or UVB (28 mJ/cm^2^) for different time periods. After incubation, cells were harvested, washed twice with PBS; and processed for RNA isolation using TRIzol method; 1 ml (5 × 10^7^ cells/ml) cell culture was pelleted down by centrifugation at 4500*g* for 10 min. The pellet was washed twice with PBS, and then 1 ml of TRIzol solution was added to each tube. After properly mixing it by pipetting, mixture was allowed to stand at room temperature for 5 min. After incubation, 0.2 ml of chloroform (100%) was added followed by vigorous shaking for 15 sec and incubation at room temperature for 3 min. Three distinct layers were visible after centrifugation at 12000*g* for 15 min at 4°C, 80% of the top layer was transferred into fresh tubes, and 0.5 ml of isopropyl alcohol (100%) was added and mixed well, incubated at room temperature for 10 min, and centrifuged at 12000*g* for 10 min at 4°C. Supernatant was discarded, and 1 ml of chilled ethanol (75%) was added to the pellet. After light vortexing, centrifugation was carried out at 7500*g* for 5 min at 4°C. Ethanol was removed carefully, and tubes were dried at room temperature for 5 min. The RNA pellet was dissolved in RNase-free double-distilled water and quantified by NanoDrop (NanoDrop 2000, Thermo Scientific, USA).

The reverse transcription reaction of mRNA was performed using Transcriptor High Fidelity cDNA Synthesis Kit as per the manufacturer's instructions. Synthesized cDNA was quantified spectrophotometrically and analysed on thermal cycler at 60°C annealing followed by 30 sec extension at 72°C (Applied Biosystems, USA) using gene-specific primers of LdUNG and *α*-tubulin whose sequences are as follows: LdUNG-For 5′ GACTCACCGCCTAAGAAACA 3′ and LdUNG-Rev 5′ AGGTCAATCGCGTCATCTC 3′; *α*-tubulin-For 5′ ATGCCAAGTGACAAGACCATTGGG 3′ and *α*-tubulin-Rev 5′ TTATTGGCAGCATCCTCCTTGCCT 3′.

### 2.10. In Vitro Methotrexate Sensitivity Assay

The effect of methotrexate (MTX) on cell growth was tested in wild-type and transfected *L. donovani* cells. All experiments with transfected cells were carried out in the presence of 0.1 mg/ml G418. Cultures containing 1 × 10^6^ cells/ml (log phase) were exposed to increasing concentrations of MTX, and cells were counted daily. IC_50_ values were obtained at the late log-phase time point as the concentration that gives 50% growth inhibition with regard to the control grown in the absence of drug and were calculated using at least five different concentrations of drug. Each value is the average of three independent experiments, and standard deviations are given.

### 2.11. DNA Fragmentation Assay

In DNA fragmentation studies, wild-type and transfected parasites from logarithmic late-phase were cultured in the presence or absence of 250 mM MTX for different durations, and approximately 1 × 10^8^ cells were harvested by centrifugation, washed twice in PBS, and incubated with a lysis buffer containing 20 mM Tris-HCl, 2 mM EDTA, and 0.2% Triton X-100, pH 7.5, for 5 min at 4°C. Lysates from the above step were centrifuged at 15,000*g*, followed by incubation of the supernatant in a solution containing 150 mM NaCl, 40 mM EDTA, 0.1% SDS, 0.2 mg/ml, 5 mg/ml RNase A, and proteinase K for 3 hours at 37°C. The fragmented nucleosomal DNA was then analysed by electrophoresis on an agarose gel [23].

### 2.12. In Vitro Drug Sensitivity Assay against AmpB and SAG

WT, UNG overexpressing, and drug-resistant parasites were treated in triplicates with varying concentrations of AmpB or SAG and checked for viability by Trypan blue dye and recording cell counts in haemocytometer. Statistical analysis was done by GraphPad Prism software v 5.0 (GraphPad Software Inc., La Jolla, CA, USA).

### 2.13. Ex Vivo Macrophage Infectivity Assessment

The isolated peritoneal macrophages from starch-induced BALB/c mice were harvested and seeded on cover slips at a density of 1 × 10^6^ cells/ml in a 6-well flat bottom culture plates in RPMI 1640 medium with 10% FBS overnight at 37°C in 5% CO_2_ incubator [24]. To remove nonadherent cells from wells, they were washed twice with PBS buffer (pH 7.2) or serum-free medium and then infection was performed with *L. donovani* promastigotes at a ratio of 10 : 1 (parasite: macrophages). After 2 hours of coincubation, noninternalized parasites were washed with RPMI 1640 medium only. Infected macrophages were further incubated for 12 and 24 hours. After 12 and 24 hours of infection, medium was removed and cells were washed twice with sterile PBS buffer (pH 7.2). Finally, cells were fixed with methanol, stained with Giemsa, and pictured with Olympus BX41 optical microscope at 100x. The numbers of infected and noninfected cells were determined, and >300 macrophages per cover slip and 100 macrophages per cover slip were counted to identify the percentage of infectivity. Further, Giemsa-stained amastigotes per 100 infected macrophages were also counted at 12 and 24 hours postinfection. The experiment was performed in triplicates, and percentage of infection is expressed as means ± SD.

### 2.14. Statistical Analysis

Statistical analysis was carried out using GraphPad Prism 5.0 software (GraphPad Software Inc., La Jolla, CA, USA). Student's *t*-test, one-way ANOVA, and mixed model ANOVA with Bonferroni posttest analysis were used to estimate the statistical significance of the differences between groups. Differences between groups were considered statistically significant when *p* value was less than 0.05 (^∗^ *p* < 0.05, ^∗∗^ *p* < 0.01, and ^∗∗∗^ *p* < 0.001).

## 3. Results

### 3.1. Partially Purified Leishmania Cell Extract Exhibits Glycosylase Activity

**Figure 1:**
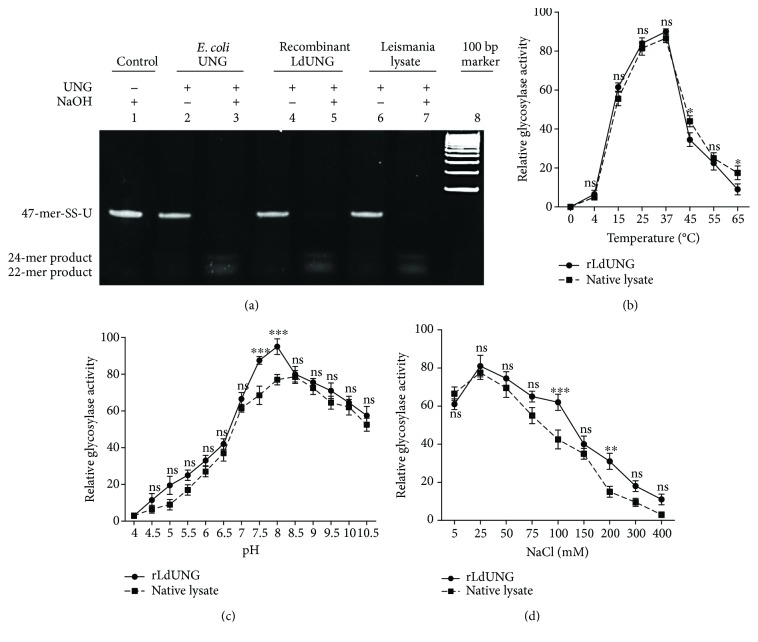
(a) Enzymatic activity of leishmania uracil DNA glycosylase. The substrate (single-stranded 47-mer oligonucleotide having uracil at the 23rd position) was treated with *E. coli* UNG, recombinant LdUNG, and parasite lysate as indicated. Lane 1, control lacking UNG; lanes 2, 4, and 6, the reactions were performed without hot alkaline (NaOH) treatment (AP lyase activity assay); lanes 3, 5, and 7, the reactions were performed with hot alkaline treatment (glycosylase activity assay). Effect of temperature (b), pH (c), and salt concentration (d) on glycosylase activity of recombinant LdUNG and native cell extract. Data was analysed using mix model ANOVA, followed by Bonferroni posttests using GraphPad Prism 5.0. Mixed model ANOVA determines how glycosylase activity of LdUNG enzyme is affected by factors (type of LdUNG (recombinant or native lysate) and different pH/temperature/salt concentration). The *p* values tell us about the difference between the groups which is likely due to the chance or due to the variable in the study. ^∗^ *p* < 0.05 means there was 5% chance that the effect may be due to random variation; ^∗∗^ *p* < 0.01 means there was 1% chance that the effect may be due to random variation; ^∗∗∗^ *p* < 0.001 means that there was 0.1% chance the effect due to random variation; ns, nonsignificant. The *y*-axis in all three graphs corresponds to relative glycosylase activity (mean ± SD) of recombinant LdUNG and leishmania lysate.

Sequence analysis of *L. donovani* genome indicated the existence of various DNA repair enzymes including UNG as described in other organisms. To check the presence of UNG activity in partially purified cell extract, removal of uracil from a specific position in the synthesized oligonucleotide-U was assayed. The *in vitro* glycosylase activity specifies the existence of UNG function in *L. donovani* (Figure 1(a)). The initial result prompted us to generate recombinant *L. donovani* UNG for further studies.

### 3.2. Cloning, Expression, and Purification of Recombinant LdUNG

UNG coding region of 1185 bp was amplified from genomic DNA of *L. donovani* and cloned into pET-28a (+) vector. The LdUNG ORF encodes for 393 amino acid long protein with predicted molecular weight of 43.2 kDa. An isoelectric point (pI) value of LdUNG was found to be 8.6. Through MITO-PROT II program and Signal IP 4.1 server, localization of LdUNG was predicted to be nuclear as well as mitochondrial.

The His-tagged LdUNG protein was expressed in *E. coli* BL21 (DE3) and purified to homogeneity using Ni^2+^-NTA agarose resins by affinity chromatography. The LdUNG-His was expressed in soluble form, and a protein yield of 0.55 mg/l of *E. coli* culture was achieved after purification. The purified LdUNG-His eluted by 100–200 mM imidazole and a single homogenous band of ~47 kDa (Figure S1) were obtained on SDS-PAGE which correlates with the predicted molecular mass of 43.24 kDa plus additional histidine tag at the N-terminus. The purified LdUNG was further used for biochemical characterization for its glycosylase activity.

### 3.3. Enzymatic Activity and Biochemical Characterization of UNG of *L. donovani*

The purified recombinant LdUNG also displayed a potent glycosylase activity by eliminating uracil from the 47-mer single-stranded oligonucleotide with U at the 23rd position (Figure 1(a)) as well as double-stranded oligonucleotide (Figure S2). When the treated abasic site containing substrate go through hot alkaline treatment, the 24-mer and 22-mer products would be generated. These products were visualized on 7 M urea 20% PAGE, and bands were found as expected. However, in the absence of hot alkaline treatment, no product was seen suggesting that the purified enzyme lacks AP lyase activity (Figure 1(a)). Therefore, LdUNG is a monofunctional enzyme. A dose and time kinetics of cleavage of uracil containing substrate by recombinant LdUNG are shown in Supplementary Figures S3 and S4. The activity was assayed at varying temperatures from 4 to 65°C, and the optimum temperature for LdUNG was found to be 37°C. The enzymatic activities decreased slowly at either lower or higher temperatures than 37°C (Figure 1(b)). The parasite cell extract displayed the glycosylase activity in the same temperature range as that of the purified recombinant enzyme. Different pH conditions also affected the activity of both purified recombinant protein and leishmania cell extract. Extract functioned in a narrow range of pH (7.5–9) compared to that of the recombinant enzyme (7.5–10.5) (Figure 1(c)). Hence, both preparations of UNG worked well under alkaline conditions. The recombinant enzyme displayed activity in a wide range of salt concentrations, that is, 0–300 mM NaCl, while more than 50% enzyme inhibition was seen at 150 mM and complete inhibition occurred at 400 mM. However, the optimal range of salt concentrations was 5–75 mM (Figure 1(d)).

The optimal salt concentration for the enzyme in lysate was 5–50 mM, whereas enzyme activity was abolished at 200 mM and above. (Figure 1(d)). However, more than 50% inhibition was found at salt range 75–100 mM, and the higher salt concentrations (150–400 mM) showed complete inhibition.

Variations in temperature, pH, and salt concentration have significant effect on the activity of any enzyme. The effects of such variations on the glycosylase activities of UNG present in recombinant purified preparation as well as in the parasite were analysed using statistical tools, that is, mixed model ANOVA followed by Bonferonni posttests. Different temperatures (*F* = 686, *p* < 0.0001), pH (*F* = 890, *p* < 0.0001), and salt concentrations (*F* = 760, *p* < 0.0001) affect the glycosylase activity of LdUNG enzyme, and the effects were found to be extremely significant. However, the effect of variations in temperature, pH, and salt concentration was found to be independent of the source (recombinant or cell lysate) of enzyme and did not show any significant difference in the effect of different temperature (*F* = 0.77, *p* = 0.47), pH (*F* = 5.54, *p* = 0.14), and salt concentration (*F* = 5.82, *p* = 0.13).

**Figure 2:**
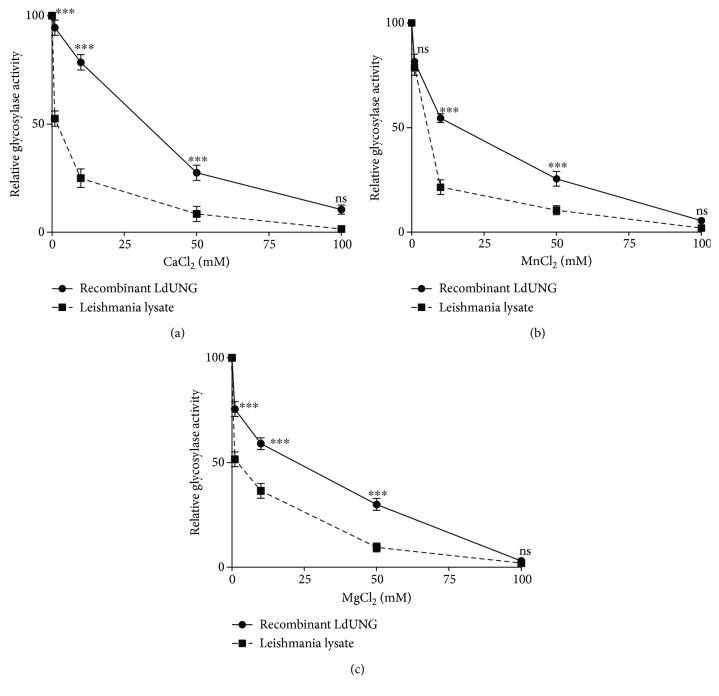
Effect of cofactors on glycosylase activity of recombinant LdUNG and native lysate assayed in the presence of 1, 10, 50, and 100 mM of CaCl_2_ (a), MnCl_2_ (b), and MgCl_2_ (c). Data was analysed using mix model ANOVA, followed by Bonferroni posttests using GraphPad Prism 5.0. Mixed model ANOVA determines how glycosylase activity of LdUNG enzyme is affected by factors (type of LdUNG (recombinant or native lysate) and different cofactors CaCl_2_/MgCl_2_/MnCl_2_). The *p* values tell us that the difference between groups is likely due to the chance or due to the variable in the study. ^∗∗∗^ *p* < 0.001 means that there was 0.1% chance that the effect may be due to random variation; ns, nonsignificant. The *y*-axis in all three graphs corresponds to relative glycosylase activity (mean ± SD) of recombinant LdUNG and leishmania lysate.

The results also indicated that the catalytic reaction of UNG did not require any cofactors *in vitro*. As indicated by mixed model ANOVA followed by Bonferroni posttests, that concentration of cofactors affects the glycosylase activity of enzyme and the effect was highly significant. Both recombinant LdUNG and leishmania lysate UNG were inhibited by increasing concentrations of cofactors such as CaCl_2_ (*F* = 951, *p* < 0.0001), MgCl_2_ (*F* = 814, *p* < 0.0001), and MnCl_2_ (*F* = 931, *p* < 0.0001) (Figure 2).

Based on kinetic parameters of recombinant LdUNG using single-stranded Uracil as substrates (Table 1), the Km value (1.24) of LdUNG was higher than that of human nuclear uracil N-glycosylase (hUNG2) [25] and *Plasmodium falciparum* PfUDG [26].

### 3.4. Overexpression of UNG Decreases Intracellular Levels of ARP under Oxidative Stress

To elucidate the role of LdUNG in survival and growth of *L. donovani* against oxidative stress, we cloned LdUNG gene into pLPneo2 vector and transfected the construct in *L. donovani* AG83 strain (henceforth designated, pLPneo2-UNG) and selected on G418. A control cell line, bearing the empty expression vector, pLPneo2, was also included. The presence of LdUNG gene in transfected parasites was confirmed by PCR (Figures S5A and S5B). Its expression was checked by real-time PCR of cDNA prepared from transfected cells (Figure S5C).

**Figure 3:**
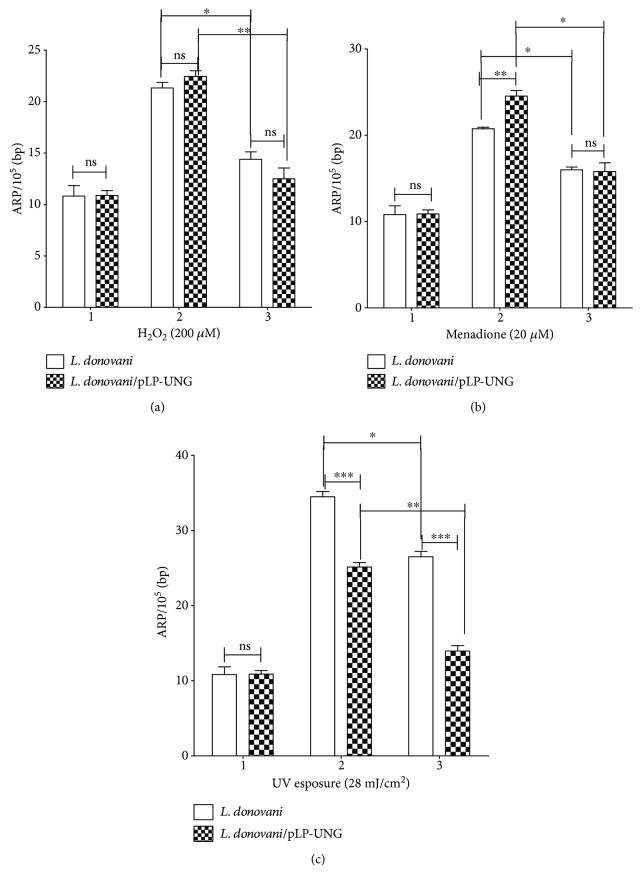
Repair of oxidative DNA damage indicated by a decrease in the number of AP sites of *L. donovani* promastigotes treated with 200 *μ*M H_2_O_2_ (a), 20 *μ*M menadione (b), and 28 mJ/cm^2^ UVB radiation (c). Generation of AP sites was evaluated by the ARP assay in wild-type and UNG overexpressing *L. donovani* strains. Levels of ARP determined in (1) untreated wild-type and UNG-OE cells and (2) wild-type and UNG-OE cells treated with oxidative agents, as indicated, for 30 min; (3) after treatment for 30 min, oxidative agents were removed by washing and the cells were allowed to recover in fresh media for 4 hours. Data were analysed using both mixed model ANOVA followed by Bonferroni posttests and one-way ANOVA test (to see differences within the group), using GraphPad Prism 5.0. Mixed model ANOVA which determines how the number of ARP sites (mean ± SD) was affected by factors (*L. donovani* strains (WT or OE) and different doses of H_2_O_2_/menadione/UVB). ^∗^ *p* < 0.05, ^∗∗^ *p* < 0.01, and ^∗∗∗^ *p* < 0.001. ns, nonsignificant. For the meaning of these statistical symbols, refer to ligand of Figure 1. The *y*-axis in all three bar graphs corresponds to the number of ARP sites per 10^5^ bp (mean ± SD) of WT and UNG-OE *L. donovani*.

Various agents that are known to induce oxidative stress increase the levels of AP sites. Though H_2_O_2_ and menadione are known ROS generators, we also estimated ROS levels in leishmania cells (~1 × 10^7^) treated with different doses of UVB radiation (302 nm) using CM-H2DCFDA as an indicator (Figure S6). Whether overexpression of UNG in *L. donovani* would enable the parasite to withstand better against the oxidative agents, we checked ARP levels in such cells and compared it with control cells that are not overexpressing (OE) UNG. The wild-type and OE cells were exposed to 200 *μ*M H_2_O_2_, 20 *μ*M menadione for 30 min, or 28 mJ/cm^2^ of UVB exposure (condition 2). Afterwards, these cells were suspended in fresh M199 medium supplemented with 10% FBS for 4 hours (condition 3). The amount of AP sites in the DNA was detected as described in Section 2.8. As expected, a significant increase was observed in damaged DNA after treatment with all oxidative agents (H_2_O_2_, menadione, and UVB) in both wild-type and OE cells (Figure 3). Upon the withdrawal of oxidative stress, the number of AP sites significantly decreased by approximately 47%, 39%, and 46% (in case of H_2_O_2_, menadione, and UVB) after 4 hours of incubation for OE cells, where in the case of wild-type cells, this decrease in AP sites was less significant with a reduction of only 30%, 22%, and 23% (in the case of H_2_O_2_, menadione, and UVB), respectively (Figure 3). A mixed model ANOVA followed by Bonferroni posttests was applied on the observed ARP levels (mean ± SD) of leishmania cells (UNG-OE and WT) at different conditions with appropriate controls. Different conditions (1, 2, and 3) had significant interaction of *L. donovani* strains after treatment with menadione (*F* = 7.73, *p* = 0.042) and UV (*F* = 62, *p* = 0.001), where the interaction was not significant after treatment with H_2_O_2_ (*F* = 5.35, *p* = 0.074). Also, *L. donovani* strains had significant effect on the results in case of treatments with menadione (*F* = 25, *p* = 0.036) or UV (*F* = 670, *p* = 0.001), whereas this effect was not significant with H_2_O_2_ (*F* = 0.19, *p* = 0.706) treatment. Different conditions also affected the ARP level, and this effect was significant for all three treatments, namely, menadione (*F* = 219, *p* < 0.001) or UV (*F* = 516, *p* < 0.0001) or H_2_O_2_ (*F* = 306, *p* = 0.0001). This indicated a clear increase in the number of healthy and apparently repaired cells in LdUNG OE parasite in comparison to wild-type cells under stress conditions as well as after rejuvenation.

### 3.5. Overexpression of LdUNG Enhances the Tolerance to Oxidative Stress Generated by Hydrogen Peroxide, UVB, Menadione, and Sodium Bisulfite

**Figure 4:**
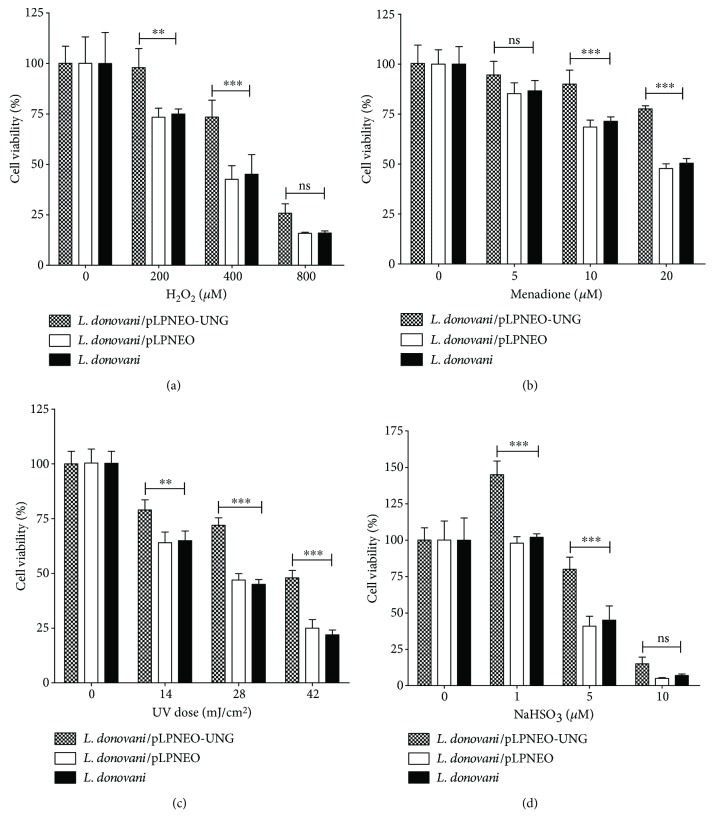
MTT-based viability assay of wild-type (*L. donovani*), LdUNG-OE (*L. donovani/*pLPneo2-UNG), and vector alone transfected (*L. donovani/*pLPneo2) strains upon treatments with H_2_O_2_ (a), menadione (b), UVB (c), and sodium bisulfite (d) at varying concentrations for a period of 1 hour followed by recovery for 4 hours in fresh media. Viability of untreated controls for each strain is taken as 100%. The results are expressed as mean ± SD of three independent experiments. Data was analysed using the mix model ANOVA, followed by Bonferroni posttests using GraphPad Prism 5.0. Mixed model ANOVA determines how the percentage viability of *L. donovani* was affected by factors (*L. donovani* strains (WT or pLPNEO-UNG or pLPNEO) and different concentrations of H_2_O_2_/menadione/UVB/NaHSO_3_), ^∗∗^ *p* < 0.01 and ^∗∗∗^ *p* < 0.001. ns, nonsignificant. For the meaning of these statistical symbols, refer to ligand of Figure 1. The *y*-axis in all four bar graphs corresponds to the percentage viability of WT/pLPNEO-UNG/pLPNEO leishmania cells (mean ± SD).

Leishmania can be cultured *in vitro* and maintained under conditions of reduced oxygen tension like many other parasitic protozoa [19]. But inside the host macrophage, the parasite faces a hostile environment with high doses of reactive oxygen species [27]. Such environment with oxidative stress may lead to DNA damage and in turn affect the cell viability. To investigate the role of UNG in protecting the parasite against oxidatively generated DNA damage, we challenged wild-type and transgenic cells with H_2_O_2_, menadione, UVB, and sodium bisulfite.  Figure 4(a) shows that H_2_O_2_ at a concentration of 200 *μ*M could reduce the viability of wild-type cells or cells transfected with the empty expression vector to 75% and 73%, respectively, but had little effect on cells overexpressing LdUNG. When used at a concentration of 400 *μ*M, H_2_O_2_ decreased the viability of wild-type cells to 42%, whereas as much as 74% of the cells overexpressing LdUNG were viable under identical condition.

Similar increase in viability was observed in transfected culture as compared to wild-type cells when both were exposed to identical concentrations of other DNA-damaging agents like menadione, UVB radiation, and sodium bisulfite (Figures 4(b)–4(d)). *L. donovani* cells overexpressing UNG displayed a 1.5- to 2-fold increase in viability compared to wild-type cells upon treatment with sodium bisulfite. An increase of 1.3–1.7-fold and 1.6–2.1-fold in survival was observed for OE parasite as compared to wild type against menadione treatment and UVB radiation, respectively. A mixed model ANOVA was applied followed by Bonferroni posttests to verify the percentage cell viability (mean ± SD, *n* = 3) of parasite cells after treatment with different oxidative agents. *L. donovani* cells (WT, vector alone, and OE) show significant effect on the percentage cell viability when treated with menadione (*F* = 25, *p* < 0.0001), UVB (*F* = 55, *p* < 0.0001), H_2_O_2_ (*F* = 14, *p* < 0.0001), and NaHSO_3_ (*F* = 30, *p* < 0.0001). Various concentrations of oxidative agents also affect the percentage cell viability, and this effect was significant for all the four treatments: menadione (*F* = 86, *p* < 0.0001), UVB (*F* = 394, *p* < 0.0001), H_2_O_2_ (*F* = 162, *p* < 0.0001), and NaHSO_3_ (*F* = 298, *p* < 0.0001). These results suggest that under oxidative stress, UNG offers a moderate protection in the parasite against oxidative DNA damage.

### 3.6. Overexpression of LdUNG Decreases Methotrexate- (MTX-) Mediated Toxicity and DNA Fragmentation

**Figure 5:**
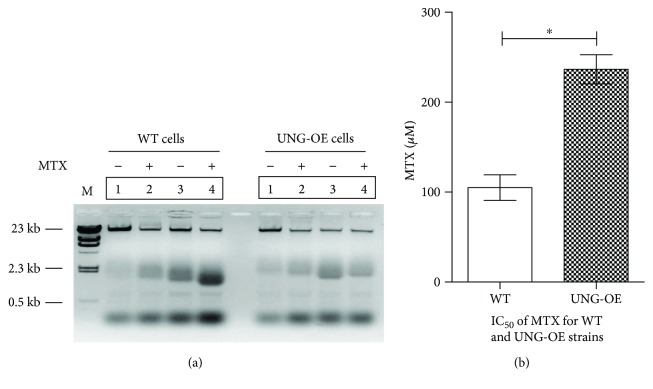
Effect of methotrexate on wild-type (WT) and UNG-OE *L. donovani* strains. (a) Total DNA isolated from WT and UNG-OE cells that were treated with 250 *μ*M methotrexate for 7 hours (lane 2) and 24 hours (lane 4). Lanes 1 and 3 correspond to untreated cells processed identically. Isolated DNA samples were analysed on 1% agarose gel stained with ethidium bromide, and the inverted picture was presented. (b) The inhibitory concentrations for MTX were determined for both WT and UNG-OE cells. Untreated parasites were used as control in this experiment. ^∗^ *p* < 0.05. ns, nonsignificant. For the meaning of these statistical symbols, refer to ligand of Figure 1. The analysis was performed by Student's *t*-tests using GraphPad Prism 5.0.

MTX is a well-known inhibitor of the de novo synthesis of thymidylate, and the outcome of this inhibition is increased levels of dUTP in the cell, which in turn leads to massive incorporation of dUTP in DNA and activation of BER [28]. The parasites overexpressing UNG show an increase in cell viability with regard to control (wild-type parasites), and IC_50_ value was found to be 2.2-fold higher than control (Figure 5(b)). Thus, at 100 *μ*M, at late log phase, growth was inhibited by 48% in control cells while only 25% loss in cell viability was observed in OE cells. As methotrexate is not involved directly in generation of ROS, but it affects indirectly by increasing dUTP level of cells and in turn increasing cell death. These results suggest a protective role of UNG against MTX-mediated toxicity.

In view of the fact that resistance to the deleterious effect of MTX was observed for *L. donovani*/pLPneo2-UNG cells, we investigated the possible association between the resistance observed and perturbation of mechanisms induced by the drug to prevent cell death. MTX is known to induce DNA fragmentation in diverse cell types when cell viability is diminished [29, 30]. To check the effect of UNG overexpression on MTX-mediated DNA fragmentation, the cells were treated with MTX and DNA was isolated and analysed by staining with ethidium bromide (Figure 5(a)). We observe a significant decrease in MTX-induced DNA fragmentation in OE parasites in comparison to WT at 7 and 24 hours after treatment. These time points were taken as in previous report, by Gallego et al. have done in *Leishmania major*.

### 3.7. Oxidative Stress Upregulates Transcription of LdUNG Gene

Treatments with agents like H_2_O_2_, menadione, and UVB increased the oxidative stress in parasite leading to activation of DNA repair pathways [31, 32]. The expression level of UNG, a crucial component of BER, was investigated. Real-time PCR analysis showed significant increase in the transcription of LdUNG gene in parasites. One way ANOVA compared the fold increase in transcriptional levels of LdUNG in the cells treated with oxidative agents for different time periods. The analysis was found to be statistically significant for H_2_O_2_ (*F* = 60, *p* < 0.0001), menadione (*F* = 32, *p* < 0.0001), and UVB (*F* = 16, *p* = 0.0010) treatments. A Tukey's multiple comparison test further indicated the significant relationship between variables (corresponding *p* values are mentioned with the readings below).

**Figure 6:**
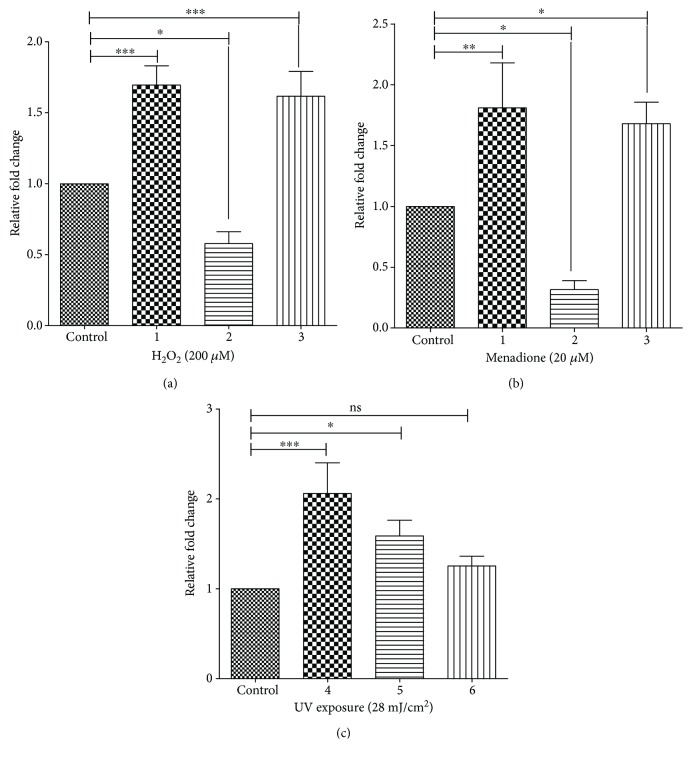
ROS inducers (H_2_O_2_, menadione, and UVB radiation) upregulate LdUNG transcription in *L. donovani*. WT cells treated with H_2_O_2_ (a), menadione (b), and UVB radiation (c). RNA level of UNG gene was checked by RTPCR in *L. donovani* treated with drug for 30 min (1), treated with the same dose for longer duration, that is, 2 hours (2), and treated with the same concentration of drug for 30 min, washed, and maintained in fresh media for 4 hours (3). In the case of UV exposure, *L. donovani* treated with 28 mJ/cm^2^ of UVB dose and RNA level of UNG gene was checked after 30 min (4), after 2 hours (5), and after 4 hours when maintained in fresh media after treatment (6). Relative abundance of UNG transcript was checked by RT-PCR, and the expression levels were normalized with that of alpha tubulin. The results are graphically represented as means ± SEM of three independent sets of experiments. Untreated parasites were used as control in the experiment. Data was analysed using one-way ANOVA, followed by Tukey's multiple comparison test using GraphPad Prism 5.0. One-way ANOVA compares the relative fold change in the transcription levels (mean) of LdUNG in four unmatched groups (control, 1/4, 2/5, and 3/6) treated with oxidative agents (H_2_O_2_/menadione/UVB). The *y*-axis in all three bar graphs corresponds to relative fold change observed in the transcription level of UNG gene in *L. donovani* cells under different treatment conditions. ^∗^ *p* < 0.05, ^∗∗^ *p* < 0.01, and ^∗∗∗^ *p* < 0.001. ns, nonsignificant. For the meaning of these statistical symbols, refer to ligand of Figure 1.

Notably, after 30 min of treatment with H_2_O_2_ and menadione, we found 1.7-fold (*p* < 0.001) and 1.81-fold (*p* < 0.01) increase in transcription of LdUNG, respectively. Moreover, the transcription of LdUNG was downregulated by 0.60- and 0.35-fold when the treatment was extended to 2 hrs. The values of upregulation of UNG gene were 1.61-fold (*p* < 0.001) and 1.68-fold (*p* < 0.05) for H_2_O_2_ and menadione, respectively, when the oxidative stress was removed after 30 min treatment and the cells were incubated for 4 hours in fresh media (Figures 6(a) and 6(b)). In case of UVB- (28 mJ/cm^2^) exposed cells, we found 2.0-fold (*p* < 0.001) and 1.65-fold (*p* < 0.05) upregulation in LdUNG transcription level after 30 min and 2 hours of incubation after radiation, respectively. The value of upregulation of UNG gene was found to be 1.25-fold in those UVB-treated cells (Figure 6(c)), which were incubated for 4 hours in fresh media after exposure. Thus, upregulated transcription of UNG lowers down as recovery proceeds. These observations suggest that with time, cells are recovering with the help of the DNA repair enzyme.

### 3.8. Overexpression of LdUNG Confers AmpB and SAG Resistance in *L. donovani*

**Figure 7:**
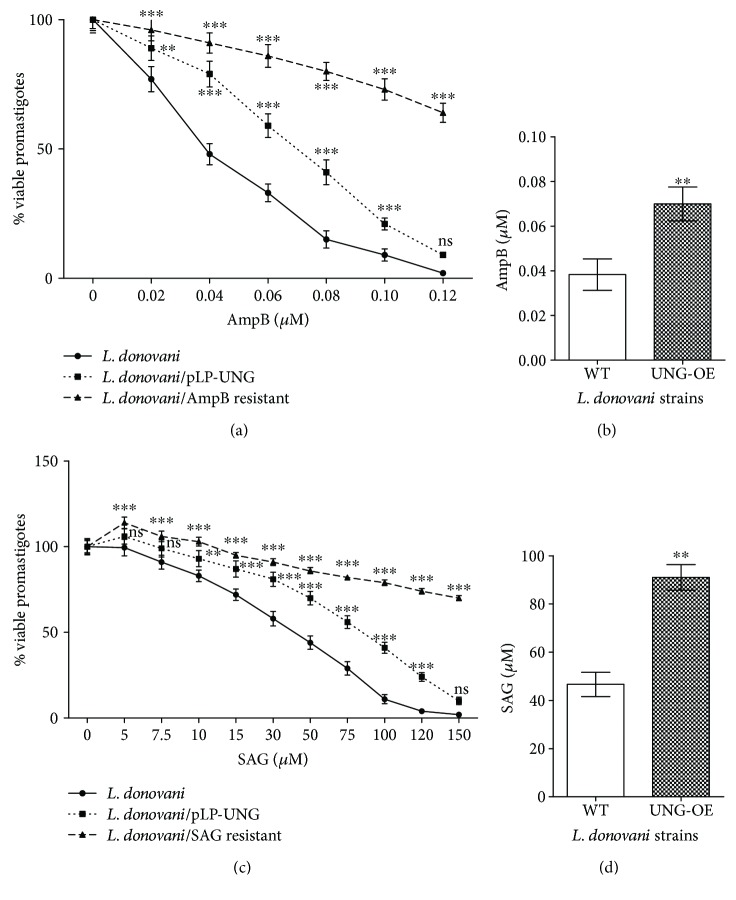
Effect of UNG overexpression on the viability of *L. donovani* in the presence of AmpB and SAG assayed by Trypan blue exclusion method. 1 × 10^6^ parasites were treated with different concentrations of AmpB (0 to 0.12 *μ*M) for 24 hours (a), and the IC_50_ for AmpB is indicated (b). Similarly, the viability with SAG treatment at 0 to 150 *μ*M (c) was determined and the IC_50_ for SAG is indicated (d). The results are expressed as means ± SD of experiments done in triplicates. Data was analysed using mix model ANOVA, followed by Bonferroni posttests (a, c) and Student's *t*-test (b, d) using GraphPad Prism 5.0. Mixed model ANOVA determines how percentage viability of promastigotes was affected by factors (*L. donovani* strains (WT or pLP-UNG or AmpB resistant) and different concentrations of AmpB/SAG). The *y*-axis in two graphs (a and c) corresponds to the percentage viability of promastigotes of WT/pLP-UNG/AmpB-resistant leishmania cells (mean ± SD). ^∗∗^ *p* < 0.01 and ^∗∗∗^ *p* < 0.001. ns, nonsignificant. For the meaning of these statistical symbols, refer to ligand of Figure 1.

Antileishmanial activities of AmpB and SAG include ROS-mediated apoptosis [33–35]. Uracil DNA glycosylase is an important BER enzyme and known to play crucial roles in the survival of organisms under stress conditions. While exploring the role of repair pathway in survival of parasite under stress conditions, we also investigated the role of UNG in drug resistance. For this, the sensitivity (IC_50_) of LdUNG overexpression (pLPneo2-UNG) and wild-type (pLPneo2 alone) parasites to AmpB and SAG was determined. It was found that pLPneo2-UNG parasites showed better survival in the presence of drugs (Figures 7(a) and 7(c)) with IC_50_ values of ~50 ng/ml and ~105 *μ*g/ml for AmpB (Figure 7(b)) and SAG (Figure 7(d)), respectively, whereas wild-type parasites showed IC_50_ of ~26 ng/ml and ~53 *μ*g/ml for AmpB and SAG, respectively. Thus, UNG overexpressing parasites exhibit 2-fold (*p* < 0.01) increase in IC_50_ for AmpB and SAG as compared to WT. As expected, treatments of AmpB and SAG had smaller effect on the cell viabilities of AmpB- and SAG-resistant strains, respectively, as compared to wild-type cells. A mixed model ANOVA followed by Bonferroni posttests was applied for the percentage of viable promastigotes (mean ± SD, *n* = 3) of different strains of *L. donovani* (WT, UNG-OE, and drug-resistant isolate), and it was found that the effects were significant for both AmpB (*F* = 458, *p* < 0.0001) and SAG (*F* = 996, *p* < 0.0001) treatments. *L. donovani* strains have highly significant interaction at all concentrations of AmpB (*F* = 41, *p* < 0.0001) and SAG (*F* = 48, *p* < 0.0001) used in this study.

### 3.9. Drug-Resistant Clinical Isolates of *L. donovani* Show Higher Levels of UNG Transcripts Compared to Sensitive Isolate

**Figure 8:**
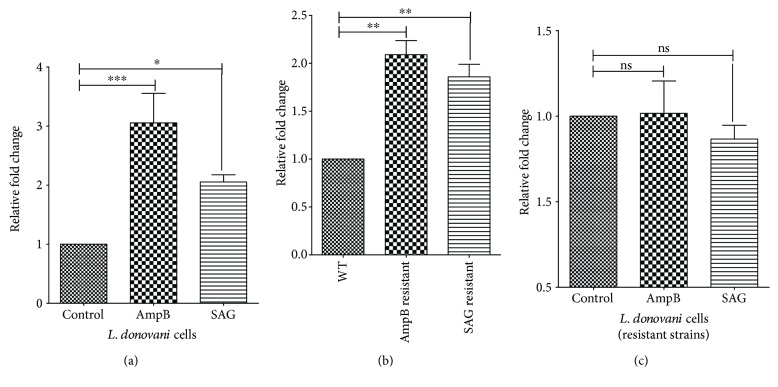
Real-time PCR showing normalized transcription level of LdUNG. (a) WT cells treated with AmpB (0.125 ng/ml) and SAG (45 *μ*M) for 8 hours. (b) Untreated WT, AmpB- and SAG-resistant clinical isolates. (c) AmpB-resistant and SAG-resistant clinical isolates treated with AmpB and SAG, respectively, for 8 hours. A housekeeping gene, *α*-Ldtubulin, was taken as internal control for normalising the variation in input. The results are expressed as mean ± SEM of three independent experiments. Data was analysed using one-way ANOVA, followed by Tukey's multiple comparison test using GraphPad Prism 5.0. One-way ANOVA compares the relative fold change of LdUNG transcription levels (mean) of three unmatched groups of leishmania cells (control, AmpB-treated/AmpB-resistant/AmpB-resistant (AmpB-treated) and SAG-treated/SAG-resistant/SAG-resistant (SAG-treated)). The *y*-axis in all three bar graphs corresponds to relative fold change observed for transcriptional level of UNG in *L. donovani* cells under different treatment conditions. ^∗^ *p* < 0.05, ^∗∗^ *p* < 0.01, and ^∗∗∗^ *p* < 0.001. ns, nonsignificant. For the meaning of these statistical symbols, refer to ligand of Figure 1.

Higher tolerance of UNG-OE parasites towards AmpB and SAG prompted us to compare the levels of UNG transcripts in drug-resistant and sensitive clinical isolates. Total RNA isolated from logarithmic phase cells were subjected to cDNA synthesis followed by real-time PCR with UNG gene-specific primers as described in Materials and Methods. As shown in Figure 8(b), the expression of LdUNG RNA was observed to be significantly higher in the resistant isolates as compared to the sensitive strain indicating an apparent association between LdUNG and drug resistance. One-way ANOVA compared the fold increase in the transcriptional levels of LdUNG in the cells resistant or sensitive to AmpB and SAG. The analysis was found to be statistically significant for both AmpB-resistant and SAG-resistant isolates (*F* = 25, *p* = 0.0012). Tukey's multiple comparison test further indicated significant relationship between variables (corresponding *p* values are given with readings below). The RT-PCR analysis showed ~2-fold (*p* < 0.01) and ~1.8-fold (*p* < 0.01) upregulation of LdUNG expression in resistant isolates (AmpB and SAG) as compared to sensitive strains in the log-phase cells. Through Western blotting using anti-LdUNG antibody, we observed similar increase in LdUNG at the protein levels in resistant and clinical isolates of the parasite (Figure S7). An upregulation of LdUNG transcription may be correlated with increase in UNG activity of resistant isolates. These results are in agreement with our observations of enhanced drug tolerance in UNG-OE parasites compared to WT.

### 3.10. Treatment with AmpB and SAG Enhances Transcription of LdUNG in Sensitive Isolates of *L. donovani*

AmpB and SAG treatments generate ROS-mediated oxidative stress in parasite which may stimulate DNA repair activities. To check whether treatment with these drugs could induce the expression of UNG in the parasite and abundance of UNG transcript was analysed by RT-PCR (Figure 8(a)). One-way ANOVA compared the fold increase in LdUNG transcripts of the parasites treated with antileishmanial drugs (AmpB and SAG). The analysis was found to be statistically significant for both AmpB- and SAG-resistant isolates (*F* = 36, *p* = 0.0004). Tukey's multiple comparison test further indicated a significant relationship between the variables (corresponding *p* values are given along with the readings below). Notably, we found ~3-fold (*p* < 0.001) and ~2-fold (*p* < 0.05) increase in expression of LdUNG at the RNA level in case of AmpB-treated and SAG-treated parasites, respectively. More interestingly, we did not find significant change in UNG transcripts when resistant strains were treated with drugs. Both AmpB- and SAG-treated resistant isolates had relatively comparable levels of UNG RNA (0.97- and 0.77-fold, resp.) which were statistically nonsignificant (*F* = 2.8, *p* = 0.135) as shown in Figure 8(c).

### 3.11. Overexpression of LdUNG in *L. donovani* Increases Infectivity

**Figure 9:**
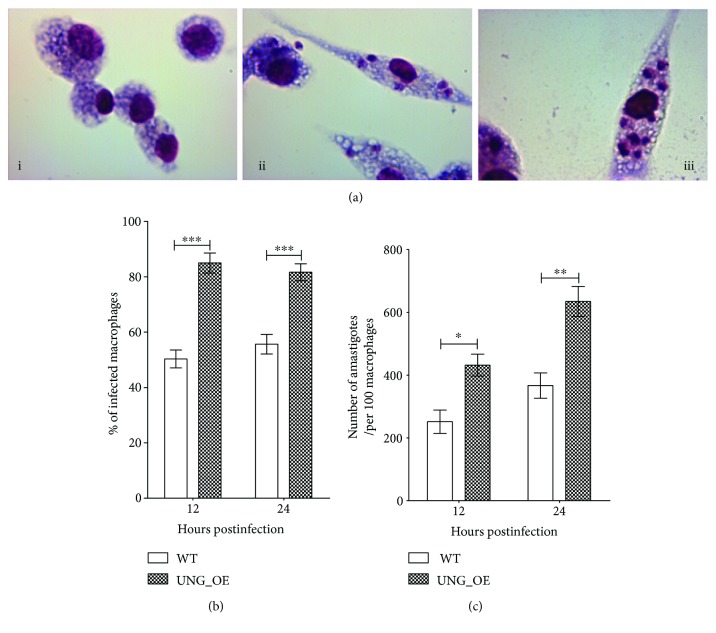
Effect of UNG overexpression on infectivity assessed by ex vivo infection in murine macrophages. (a) Microscopic pictures of Giemsa-stained uninfected macrophages (i), macrophages infected with WT (ii), and UNG-OE (iii), respectively. (b) Percentage of infected macrophages determined at 12 and 24 hours postinfection. (c) Number of amastigotes per 100 infected macrophages recorded at 12 and 24 hours postinfection. The results are expressed as mean ± SD of three independent experiments. Data was analysed using mix model ANOVA, followed by Bonferroni posttests using GraphPad Prism 5.0. Mixed model ANOVA determines how percentage of infected macrophages/number of amastigotes per 100 macrophages was affected by different factors (*L. donovani* strains, WT or UNG-OE, and different time intervals of 12 and 24 hours postinfection). The *y*-axis in two bar graphs corresponds to percentage of infected macrophages/number of amastigotes per 100 macrophages of WT/UNG-OE leishmania cells. ^∗^ *p* < 0.05, ^∗∗^ *p* < 0.01, and ^∗∗∗^ *p* < 0.001. ns, nonsignificant. For the meaning of these statistical symbols, refer to ligand of Figure 1.

Oxidative stress present in host macrophage poses genotoxic threat to the parasite. Overexpression of UNG might present an advantage for the parasite in the processes and establishment of infection. To corroborate this, we compared the infectivity of wild-type and overexpressed parasites in macrophages using an ex vivo model. This helps us to elucidate the role of LdUNG in counteracting the oxidative hostile conditions inside the human macrophage during infection. Peritoneal macrophages isolated from starch-induced BALB/c mice were infected with wild-type and LdUNG-OE parasites. A set of uninfected macrophages (Figure 9) was kept as control for comparison. Infected or control cells were stained with Giemsa for microscopic examination. The pictures of uninfected macrophages (i), macrophages infected with *L. donovani* (ii), and LdUNG overexpressed *L. donovani* (iii) parasites (Figure 9(a)) showed that parasites overexpressing LdUNG exhibit more infection as well as number of amastigotes per macrophages as compared to wild-type parasites. By counting infected and noninfected macrophages, we compared the infectivity and infection rate of both types of parasite. A significantly higher (~1.5-fold, *p* < 0.01) percentage of macrophage infectivity (~81%) was observed in transfected parasites as compared to wild-type parasites (~55%) (Figure 9(b)). A mixed model analysis of variance followed by Bonferroni posttests verified the percentage of infected macrophages (mean ± SD, *n* = 3) of different parasite strains. *L. donovani* cells (WT and UNG-OE) affects the percentage of infected macrophage, and the effect was considered significant (*F* = 245, *p* < 0.0001). *L. donovani* strains did not have significant interaction at all the values of time (*F* = 5.02, *p* = 0.055). Different time periods (12 hr and 24 hr) did not affect the percentage of infected macrophages, and this effect was considered nonsignificant (*F* = 0.26, *p* = 0.620).

The number of amastigotes per infected macrophage was also increased supporting the increased infectivity percentage. After 12 hours of infection with wild-type and LdUNG parasites, numbers of amastigotes per 100 infected macrophages were found to be 252 ± 37 and 432 ± 35, respectively. Similarly, after 24 hours of infection with wild-type and OE parasites, numbers of amastigotes per 100 infected macrophages were found to be 367 ± 40 and 635 ± 54, respectively (Figure 9(c)). Thus, the transfected parasite showed a significant ~1.8-fold (*p* < 0.01) higher number of amastigotes per infected macrophage when compared to wild type. Our results show that from 12 hours to 24 hours' time period, the parasites did not show significant change except 10–15% increase in number of amastigotes. A mixed model ANOVA followed by Bonferroni posttests was applied to analyse the number of amastigotes per 100 macrophages (mean ± SD, *n* = 3) of different parasite strains. *L. donovani* cells (WT and UNG-OE) significantly (*F* = 61, *p* = 0.0014) affects the number of amastigotes per 100 macrophages. *L. donovani* strains did not have significant interaction at all values of time (*F* = 2.38, *p* = 0.197). Different time periods (12 and 24 hours) affect the number of amastigotes per 100 macrophages, and this effect was considered to be significant (*F* = 31.12, *p* = 0.0051). These results demonstrate that LdUNG overexpression increases the infectivity of *L. donovani* parasites suggesting its potential role in survival under oxidative stress conditions inside macrophages.

## 4. Discussion

DNA repair pathways play an important role in sustaining and continuity of life by correcting the anomalies in the genomic DNA. They provide protection against different types of endogenous and exogenous sources of DNA damage. In other words, they help organisms in evading the ill effects of stressful and extreme environments. The prominence of stress management is one of the vital survival strategies in every organism's life to exist in this world. The same is the case with *Leishmania donovani*, a protozoan parasite which causes lethal disease visceral leishmaniasis. After going through different aspects of this parasite, it seems that its development, infectivity, and pathogenesis are chiefly related to the parasite stress response. The complex interactions between parasite stress response, cell cycle regulation, and differentiation into various life cycle stages [36] help the parasite to cause infection by acclimatizing to the oxidative and nitrosative bursts during host-pathogen interaction and establishment of infection [37, 38]. The cellular-derived ROS production within the cell is majorly due to NADPH oxidase releasing superoxide ion (O_2_^•−^). This superoxide ion reacts with NO to form peroxynitrite (ONOO^−^) and also forms more damaging hydroxyl radical (^•^OH) due to change in iron radicals, Haber-Weiss and Fenton chemistry [39]. The ROS-generated oxidative stress leads to DNA damage ultimately causing cell death and tissue damage [40]. The lesions generated in DNA due to ROS are mainly repaired by BER, making the pathway indispensable for leishmania to persist inside the host macrophage. UNG is one of the important components of this pathway solely for its upfront capability of detecting and isolating the lesions. Therefore, appropriate functioning of UNG is of paramount importance for maintenance of BER pathway to repair DNA lesions and help the parasite in combating with the deleterious effects of ROS. In the present study, we demonstrate the role of LdUNG in protection against DNA-damaging agents and oxidative stress. Apart from this, we show that UNG is indirectly related to the mechanisms involving drug resistance in leishmania. First, we demonstrated the presence of glycosylase activity in leishmania lysate and then in heterologously produced and purified recombinant LdUNG. We find that overexpression of enzyme in the parasite confers tolerance against oxidative stress by correcting DNA damage and surprisingly against antileishmanial drugs which are in use. Also, our studies revealed that stressed parasites or resistant isolates exhibit upregulated levels LdUNG transcription. Interestingly, overexpression of UNG enhances the infectivity of the parasite.

To date, many UDGs from different organisms have been characterized for their biochemical properties like the bacterial enzyme (*Escherichia coli* [41, 42]), viral enzymes (poxvirus [43] and HSV-1 [44]), parasite enzyme (*Trypanosoma cruzi* [45] and *Plasmodium falciparum* [26]), and human enzyme [25, 46, 47].

We characterized the enzyme biochemically on the basis of its glycosylase activity of nicking uracil out from a specific position in synthetic oligonucleotide. Both native and recombinant forms of LdUNG are sensitive towards change in temperature and are best active at optimum temperature 37°C, which is also a physiological temperature of the human host (Figure 1(b)). The other reported UDGs also work at the same optimal temperature except for *T. cruzi* which works optimally at 45°C [45]. The alkaline range of pH 7–9 is best suited for the enzyme functionality (Figure 1(c)), and this is in accordance with other reported UDGs [41, 45, 47]. The activity of parasitic glycosylase ceases at higher salt concentrations, and 50–100 mM was the optimum concentration (Figure 1(d)), which is in accordance with other reported 50 mM in hUNG [47], 65 mM in *T. cruzi* UDG [45], and 50–75 mM in *P. falciparum* UNG [26]. Most of the enzymes lose activity in higher concentrations of neutral salts due to high ionic strength produced [48]. For understanding requirements of cofactors, recombinant purified LdUNG and cell lysate both showed good activity in the absence of any external divalent cation cofactors including Mg^2+^, Mn^2+^, and Ca^2+^ (Figure 2). Our results are in agreement with those for *E. coli* UNG and *P. falciparum* UNG [26] that did not require any cofactors for their function. However, other UDGs like human [47], vaccinia virus [49], and HSV-2 [50] showed activity with optimal MgCl_2_ at 6 mM, 7.5 mM, and 5 mM, respectively. Therefore, LdUNG is advocated as a cofactor-independent enzyme which is different from the human enzyme. The structural changes or direct interactions with the negatively charged group of the enzyme are suggested to be induced by high concentrations of divalent cations. The recombinant LdUNG showed tolerance towards a broader range of assay conditions as compared to parasite lysate under identical assay conditions.

The role of UNG in survival and growth of organisms under oxidative stress has not been studied in detail. The role of UNG enzyme in repair of oxidative damage has been described in human [51]. To elucidate the role of UNG in survival under oxidative stress, we overexpressed it by transfecting *L. donovani* cells with pLPneo2-LdUNG plasmid. Our results revealed that overexpression of UNG conferred significant resistance to the oxidative stress through direct production of ROS by agents like hydrogen peroxide and menadione or similar stress indirectly using UVB radiation and sodium bisulphite (Figure 4). The H_2_O_2_ and menadione induce ROS-mediated killing of both wild-type and UNG-OE cells, but overexpressed cells are better protected against oxidative killing as compared to wild type. Our findings supported by other reports like overexpression of UNG1 enhanced cell resistance to oxidative stress and protected mitochondrial DNA from oxidation [52]. It was also reported that hydrogen peroxide results in activation of diverse cellular pathways including DNA repair [31]. In an opposite direction, 8-oxoguanine DNA glycosylase 1 (Ogg1) when overexpressed in *Trypanosoma cruzi* increases its sensitivity towards hydrogen peroxide, and the low turnover number of Ogg1 compared to other DNA glycosylases might be the reason for the increased sensitivity [53]. Other DNA-damaging agents like UV radiation also generate ROS [54] and induce a better stress response in the UNG-OE cells as compared to wild-type cells. Sodium bisulfite is mainly responsible for the incorporation of uracil in the genomic DNA which leads to DNA damage [55], and uracil misincorporation was basically corrected by UNG. The UNG-OE parasite survives better when treated with different doses of sodium bisulfite as compared to wild-type cells.

Methotrexate is a non-ROS-generating agent, but it increases the intracellular levels of dUTP due to inhibition of dTTP synthesis resulting from inhibition of dihydrofolate reductase [22]. This increased dUTP allows incorporation of uracil into DNA during replication. When *L. donovani* cells were challenged with methotrexate, increased level of LdUNG in OE cells results in higher rate of dUTP elimination from DNA; therefore, increased resistance to MTX was observed (Figure 5). In earlier report, advantage in survival for APE overexpressing *L. major* against methotrexate was described [56].

Further, through an indirect assay, we quantified the levels of AP sites using ARP in parasites and found that the number of oxidative lesions was increased when cells were exposed to stress environment. The same type of observation was reported in *T. cruzi*, where DNA damage and repair were quantified against oxidative agents like ROS and iNOS by detecting levels of AP sites [57]. The UNG-OE parasite displayed significant decrease in the AP sites as compared to wild-type parasite when exposed to oxidative environment (Figure 3). After removal of stress, number of AP sites decreases with time in both wild-type and overexpressed cells. It indicates the recovery done by repair pathways against the damage generated during the stress conditions.

The change in gene expression levels due to exposure to oxidative stress form an important part of cellular defence mechanism and is proposed to be achieved at posttranscriptional level in leishmania [58]. An upregulation of LdUNG and other components of repair pathway is induced in response to oxidative stress and is maintained during recovery even after removal of stress. Previously, upregulation of DNA repair genes was reported in response to hydrogen peroxide in human [59], expression of Ntg1, and a DNA repair gene in *Saccharomyces cerevisiae* is induced upon oxidative stress and it is localized to both the nucleus and mitochondria [60]. Better survival of human macrophage in high ROS condition due to increased expression DNA repair protein was hypothesized earlier [61]. Our findings also fall in the same line, and an upregulation of LdUNG is seen when parasite is treated with ROS-generating oxidants like H_2_O_2_ and menadione as well UVB radiation (Figure 6). The level of expression was significantly downregulated when cells were kept under stress condition for a longer period (Figure 6). It was evident that prolonged exposure to stress condition might arrest cell cycle resulting in observed decrease in expression of repair genes [62]. Elevated expression of LdUNG was more or less maintained even after the stress (H_2_O_2_ and menadione) was withdrawn and cells were grown in fresh media for four hours. In this context, the observed upregulation of UNG transcription in parasites on treatment with ROS inducers supports the established role of the UNG repair protein in stress tolerance. UVB-induced upregulation decreased at four hours of postexposure recovery, though levels of UNG were more in comparison to unexposed control.

Most antileishmanials are reported to induce apoptosis-associated features in leishmania, which include increase in intracellular calcium levels, accumulation of ROS, drop of the mitochondrial membrane potential (Dcm), genomic DNA degradation, exposure of phosphatidylserine, and induction of caspase-like activity [63–67]. Hence, the killing mechanisms of drugs AmpB and SAG include cell apoptosis by accumulation of ROS. According to previous report, promastigotes of sensitive strain of *L. infantum* showed high levels of ROS in response to antimonial (SbIII), miltefosine, and AmpB treatments [63]. Therefore, it was interesting to investigate whether overexpression of UNG could have an effect on survival of *L. donovani* upon treatment with AmpB and SAG and we find that the overexpression helps parasite to survive better. Further, IC_50_ doses of AmpB and SAG drugs induce ~3- and ~2-fold increase in UNG transcription in *L. donovani*, respectively (Figure 7). After getting increased transcription of repair gene in drug-treated parasite, we were curious to compare the expression levels of UNG in drug-resistant and sensitive isolates of parasite. The AmpB- and SAG-resistant strains of parasite displayed significant increase in the transcripts of the UNG gene (Figure 8). These findings were supported by the report of upregulated expression of RAD51, a DNA repair enzyme, being involved in drug resistance in trypanosomatids [68]. Resistance is accompanied by tolerance to drug-induced apoptosis not only against the selective drug but also against drugs sharing a similar mode of killing [69]. We further treated drug-resistant strains of the parasite with respective drugs, that is, AmpB and SAG. No significant change was observed in the expression level of UNG in the drug-treated resistant parasite (Figure 8(c)). The finding was supported by a report which demonstrate the success of resistant strain in avoiding oxidative stress in *L. infantum* [70], and parasite field isolates resistant to SbIII were less prone to undergo apoptosis following exposure to either SbIII, miltefosine, or AmpB [63]. Resistance mechanisms involving DNA repair proteins may encompass point mutations and modulation of parasitic gene expression by the amplification or deletion of key genes involved in resistance. This phenomenon of gene rearrangement is particularly prevalent in the genus Leishmania and is often observed in drug-resistant mutants [71].

An increase in stress would be an imperative imposition on stationary stage parasites because being the preparative phase before infection, the parasites need to armour themselves with efficient redox detoxification machinery to promptly counteract the oxidative/nitrosative burst and hostile conditions inside the macrophage during infection. In this perspective, our ex vivo infectivity studies of wild-type and UNG-OE parasites (Figure 9) demonstrated that percentage of infectivity and number of amastigotes per infected macrophage were considerably higher with UNG-OE parasite as compared to wild type.

## 5. Conclusion

**Figure 10:**
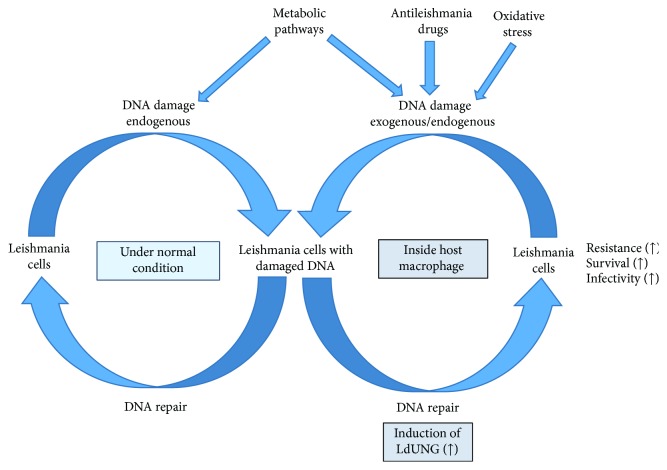
Graphical representation.

Considering the persistence of parasite in macrophage cells of host is responsible for the chronic phase of visceral leishmaniasis, our results suggest that LdUNG plays a key role in BER in *L. donovani* and its overexpression confers moderate resistance to oxidants. The UNG protein was upregulated in stress conditions. The effect of stress from both exogenous and endogenous sources on the induction of DNA repair genes, especially UNG, is represented in graphical illustration in Figure 10. Our results emphasize the role of BER pathway in establishment of infection in human macrophage. Added to this, association of UNG expression with resistance against commonly used antileishmanial drugs opens a new window of opportunity to target LdUNG for development of combination therapy against leishmaniasis. Thus, drug combinations for the treatment of leishmaniasis represent a promising area of research, and as such, targeting DNA repair in combination with other drugs in practice should be considered. The combination of therapeutic arsenals to increase treatment efficiency as well as decrease cure duration and drug resistance is the most promising strategy, since no vaccine against trypanosomatids has been discovered so far [72].

## Figures and Tables

**Table 1:** Comparison of kinetic parameter of recombinant UNG of leishmania with that of plasmodium and human.

Substrate	Km (*μ*M)		
	LdUNG	PfUDG	hUNG
47-mer-SS-U	1.24 ± 0.9	1.63^∗^	2.2 ± 0.2^∗^

^∗^Suksangpleng et al. [26].

## Supplementary Materials

Supplementary Materials Figure S1: analysis of recombinant LdUNG expression by 12% SDS and Western blot. (A) SDS-PAGE: M, unstained protein marker; lane 1, uninduced supernatant; lane 2, induced pellet fraction (IPTG 0.2 mM); lane 3, induced supernatant fraction (IPTG 0.2 mM); lane 4, induced pellet fraction (IPTG 0.4 mM); lane 5, induced supernatant fraction (IPTG 0.4 mM); lane 6, induced pellet fraction (IPTG 0.6 mM); and lane 7, uninduced supernatant fraction. (B) Western blot: lane 3, purified LdUNG; primary Ab, anti-His (dilution 1 : 12000); secondary Ab, anti-mouse conjugated with HRP (1 : 15000). Figure S2: enzymatic activity of recombinant LdUNG on single- and double-stranded substrates. Figure S3: dose-dependent enzymatic activity of leishmania uracil DNA glycosylase. The substrate (single-stranded 47-mer oligonucleotide having uracil at 23 position) was treated with recombinant LdUNG (A) and leishmania lysate (B) as indicated. Figure S4: time-dependent enzymatic activity of leishmania uracil DNA glycosylase. The substrate (single-stranded 47-mer oligonucleotide having uracil at the 23rd position) was treated with recombinant LdUNG for different incubation time as indicated. Figure S5: (A) schematic diagram showing primers used for confirmation of clone in transfected *L. donovani*. For_v indicates vector-specific forward primer. For_g and Rev_g indicate gene-specific forward and reverse primers, respectively. (B) Confirmation of LdUNG-transfected *L. donovani* by PCR. Lane 1 indicates 100 bp DNA ladder. Lanes 2 and 3 represent PCR amplification using plasmid isolated from LdUNG transfectants. Lanes 4 and 5 contain PCR products amplified using vector pLPneo2 DNA. Lanes 6 and 7 contain PCR amplification using *L. donovani*-genomic DNA as template. Lanes 3, 5, and 7 are amplification products with vector-specific forward and gene-specific reverse primers. Lanes 2, 4, and 6 represent PCR products using gene-specific forward and reverse primers. (C) Relative abundance of UNG transcripts in WT and LdUNG transfectants determined by RT-PCR taking *α*-tubulin as internal control. Figure S6: estimation of ROS in UV-exposed *L. donovani* promastigotes. ROS was estimated in parasite cells after 30 min post UV (302 nm) treatment using CM-H2DCFDA as indicator. Figure S7: Western blot. Total cell lysates prepared from sensitive, AmpB-resistant, and SAG-resistant *L. donovani* strains were resolved on 10% SDS-PAGE and subjected to Western blot analysis using LdUNG and *α*-LdActin antibodies, respectively. Primary Ab: anti-LdUNG (dilution 1 : 5000); secondary Ab: anti-rabbit conjugated with HRP (1 : 15000). Table S1: sequences of oligonucleotides used as substrates in UNG activity assay.

## References

[1] Chappuis F., Sundar S., Hailu A. (2007). Visceral leishmaniasis: what are the needs for diagnosis, treatment and control?. *Nature Reviews Microbiology*.

[2] Alvar J., Vélez I. D., Bern C. (2012). Leishmaniasis worldwide and global estimates of its incidence. *PLoS One*.

[3] Lawyer P. G., Perkins P. V. (2004). Leishmaniasis and trypanosomiasis. *Medical Entomology*.

[4] WHO Fact Sheet Leishmaniasis. http://www.who.int/mediacentre/factsheets/fs375/en/.

[5] Wasan K. M., Wasan E. K., Gershkovich P. (2009). Highly effective oral amphotericin B formulation against murine visceral leishmaniasis. *The Journal of Infectious Diseases*.

[6] Monge-Maillo B., Lopez-Velez R. (2013). Therapeutic options for visceral leishmaniasis. *Drugs*.

[7] Sundar S., Sinha P. K., Rai M. (2011). Comparison of short-course multidrug treatment with standard therapy for visceral leishmaniasis in India: an open-label, non-inferiority, randomized controlled trial. *The Lancet*.

[8] Chakarvarty J., Sundar S. (2010). Drug resistance in leishmaniasis. *Journal of Global Infectious Diseases*.

[9] Molyneux D., Killick-Kendrick R., Peters W., Killick-Kendrick R. (1987). Morphology, ultrastructure and lifecycles. *Leishmaniasis in Biology and Medicine*.

[10] Mauël J. (1996). Intracellular survival of protozoan parasites with special reference to *Leishmania* spp., *Toxoplasma gondii* and *Trypanosoma cruzi*. *Advances in Parasitology*.

[11] Desjardins M., Descoteaux A. (1997). Inhibition of phagolysosomal biogenesis by the leishmania lipophosphoglycan. *Journal of Experimental Medicine*.

[12] Moore K. J., Matlashewski G. (1994). Intracellular infection by *Leishmania donovani* inhibits macrophage apoptosis. *The Journal of Immunology*.

[13] Bogdan C., Rollinghoff M. (1998). The immune response to *Leishmania*: mechanisms of parasite control and evasion. *International Journal for Parasitology*.

[14] Kaye P. M. (1995). Costimulation and the regulation of antimicrobial immunity. *Immunology Today*.

[15] McMahon-Pratt D., Kima P. E., Soong L. (1998). Leishmania amastigote target antigens: the challenge of a stealthy intracellular parasite. *Parasitology Today*.

[16] Cadet J., KJA D. (2017). Oxidative DNA damage & repair: an introduction. *Free Radical Biology & Medicine*.

[17] Nicholl I. D., Nealon K., Kenny M. K. (1997). Reconstitution of human base excision repair with purified proteins. *Biochemistry*.

[18] Nilsen H. (2002). DNA base excision repair of uracil residues in reconstituted nucleosome core particles. *The EMBO Journal*.

[19] Mehlotra R. K. (1996). Antioxidant defense mechanisms in parasitic protozoa. *Critical Reviews in Microbiology*.

[20] Visnes T., Akbari M., Hagen L., Slupphaug G., Krokan H. E. (2008). The rate of base excision repair of uracil is controlled by the initiating glycosylase. *DNA Repair*.

[21] Jha P. K., Khan M. I., Mishra A., Das P., Sinha K. K. (2017). HAT2 mediates histone H4K4 acetylation and affects micrococcal nuclease sensitivity of chromatin in *Leishmania donovani*. *PLoS One*.

[22] Singh J., Srivastava A., Jha P., Sinha K. K., Kundu B. (2015). L-Asparaginase as a new molecular target against leishmaniasis: insights into the mechanism of action and structure-based inhibitor design. *Molecular BioSystems*.

[23] Holzmuller P., Sereno D., Cavaleyra M. (2002). Nitric oxide-mediated proteasome-dependent oligonucleosomal DNA fragmentation in *Leishmania amazonensis* Amastigotes. *Infection and Immunity*.

[24] Ray A., Dittel B. N. (2010). Isolation of mouse peritoneal cavity cells. *Journal of Visualized Experiments*.

[25] Kavli B., Sundheim O., Akbari M. (2002). hUNG2 is the major repair enzyme for removal of uracil from U: A matches, U: G mismatches, and U in single-stranded DNA, with hSMUG1 as a broad specificity backup. *Journal of Biological Chemistry*.

[26] Suksangpleng T., Leartsakulpanich U., Moonsom S. (2014). Molecular characterization of *Plasmodium falciparum* uracil-DNA glycosylase and its potential as a new anti-malarial drug target. *Malaria Journal*.

[27] Klebanoff S. J. (1980). Oxygen metabolism and the toxic properties of phagocytes. *Annals of Internal Medicine*.

[28] Ladner R. D. (2001). The role of dUTPase and uracil-DNA repair in cancer chemotherapy. *Current Protein & Peptide Science*.

[29] Heenen M., Laporte M., Noel J. C., de Graef C. (1998). Methotrexate induces apoptotic cell death in human keratinocytes. *Archives of Dermatological Research*.

[30] Govlian M., Bleile B., Tseng B. Y. (1980). The effect of methotrexate on levels of dUTP in animal cells. *Journal of Biological Chemistry*.

[31] Bigarella C. L., Liang R., Ghaffari S. (2014). Stem cells and the impact of ROS signaling. *Development*.

[32] Schucha A. P., Morenob N. C., Schuchc N. J., Menck C., Camila Garcia C. C. (2017). Sunlight damage to cellular DNA: focus on oxidatively generated lesions. *Free Radical Biology & Medicine*.

[33] Singh K., Garg G., Ali V. (2016). Current therapeutics, their problems and thiol metabolism as potential drug targets in leishmaniasis. *Current Drug Metabolism*.

[34] Brajtburg J., Powderly W. G., Kobayashi G. S., Medoff G. (1990). Amphotericin B: current understanding of mechanisms of action. *Antimicrobial Agents and Chemotherapy*.

[35] No J. H. (2016). Visceral leishmaniasis: revisiting current treatments and approaches for future discoveries. *Acta Tropica*.

[36] Zilberstein D., Shapira M. (1994). The role of pH and temperature in the development of *Leishmania* parasites. *Annual Review of Microbiology*.

[37] Van A. T., Deschacht M., Da L. R., Maes L., Cos P. (2011). *Leishmania*-macrophage interactions: insights into the redox biology. *Free Radical Biology & Medicine*.

[38] Deschacht M., Van A. T., Hendrickx S., Bult H., Maes L., Cos P. (2012). Role of oxidative stress and apoptosis in the cellular response of murine macrophages upon *Leishmania* infection. *Parasitology*.

[39] Kirkham P. (2007). Oxidative stress and macrophage function: a failure to resolve the inflammatory response. *Biochemical Society Transactions*.

[40] YueTan H., Wang N., Li S., Hong M., Wang X., Feng Y. (2016). The reactive oxygen species in macrophage polarization: reflecting its dual role in progression and treatment of human diseases. *Oxidative Medicine and Cellular Longevity*.

[41] Lindahl T. (1974). An *N*-glycosidase from *Escherichia coli* that releases free uracil from DNA containing deaminated cytosine residues. *Proceedings of the National Academy of Sciences of the United States of America*.

[42] Lindahl T., Ljungquist S., Siegert W., Nyberg B., Sperens B. (1977). DNA N-glycosidases: properties of uracil-DNA glycosidase from *Escherichia coli*. *Journal of Biological Chemistry*.

[43] Stuart D. T., Upton C., Higman M. A., Niles E. G., McFadden G. (1993). A poxvirus-encoded uracil DNA glycosylase is essential for virus viability. *Journal of Virology*.

[44] Argnani R., Focher F., Zucchini S. (1995). Herpes simplex virus type 1 (HSV-1) uracil-DNA glycosylase: functional expression in *Escherichia coli*, biochemical characterization, and selective inhibition by 6-(*p-n*-octylanilino)uracil. *Virology*.

[45] Peña-Diaz J., Akbari M., Sundheim O. (2004). *Trypanosoma cruzi* contains a single detectable uracil-DNA glycosylase and repairs uracil exclusively via short patch base excision repair. *Journal of Molecular Biology*.

[46] Slupphaug G., Oisen L. C., Helland D., Aasland R., Krokan H. E. (1991). Cell cycle regulation and *in vitro* hybrid arrest analysis of the major human uracil-DNA glycosylase. *Nucleic Acids Research*.

[47] Visnes T., Doseth B., Pettersen H. S. (2009). Uracil in DNA and its processing by different DNA glycosylases. *Philosophical Transactions of the Royal Society B*.

[48] Warren J. C., Stowring L., Morales M. F. (1966). The effect of structure-disrupting ions on the activity of myosin and other enzymes. *The Journal of Biological Chemistry*.

[49] Scaramozzino N., Sanz G., Crance J. M. (2003). Characterisation of the substrate specificity of homogeneous vaccinia virus uracil-DNA glycosylase. *Nucleic Acids Research*.

[50] Winters T. A., Williams M. V. (1993). Purification and characterization of the herpes simplex virus type 2-encoded uracil-DNA glycosylase. *Virology*.

[51] Liu Z., Hu Y., Gong Y., Liu C., Wang Q. (2016). Hydrogen peroxide mediated mitochondrial UNG1-PRDX3 interaction and UNG1 degradation. *Free Radical Biology & Medicine*.

[52] Dizdaroglu M., Karakaya A., Jaruga P., Slupphaug G., Krokan H. E. (1996). Novel activities of human uracil DNA N-glycosylase for cytosine-derived products of oxidative DNA damage. *Nucleic Acids Research*.

[53] Furtado C., Kunrath-Lima M., Rajão M. A. (2012). Functional characterization of 8-oxoguanine DNA glycosylase of *Trypanosoma cruzi*. *PLoS One*.

[54] Pattison D. I., Davies M. J. (2006). Actions of ultraviolet light on cellular structures. *Experientia Supplementum*.

[55] Kato Y., Moriwaki T., Funakoshi M., Zhang-Akiyama Q. M. (2015). *Caenorhabditis elegans* EXO-3 contributes to longevity and reproduction: differential roles in somatic cells and germ cells. *Mutation Research/Fundamental and Molecular Mechanisms of Mutagenesis*.

[56] Gallego C., Estevez A. M., Fárez E., Ruiz-Pérez L. M., González-Pacanowska D. (2005). Overexpression of AP endonuclease protects *Leishmania major* cells against methotrexate induced DNA fragmentation and hydrogen peroxide. *Molecular and Biochemical Parasitology*.

[57] Cabrera G., Barria C., Fernández C. (2011). DNA repair BER pathway inhibition increases cell death caused by oxidative DNA damage in *Trypanosoma cruzi*. *Journal of Cellular Biochemistry*.

[58] Vanhamme L., Pays E. (1995). Control of gene expression in trypanosomes. *Microbiological Reviews*.

[59] Santa-Gonzalez G. A., Gomez-Molina A., Arcos-Burgos M., Meyer J. N., Camargo M. (2016). Distinctive adaptive response to repeated exposure to hydrogen peroxide associated with upregulation of DNA repair genes and cell cycle arrest. *Redox Biology*.

[60] You H. J., Swanson R. L., Harrington C. (1999). *Saccharomyces cerevisiae* Ntg1p and Ntg2p: broad specificity N-glycosylases for the repair of oxidative DNA damage in the nucleus and mitochondria. *Biochemistry*.

[61] Bauer M., Goldstein M., Christmann M., Becker H., Heylmann D., Kaina B. (2011). Human monocytes are severely impaired in base and DNA double-strand break repair that renders them vulnerable to oxidative stress. *Proceedings of the National Academy of Sciences of the United States of America*.

[62] Dimozi A., Mavrogonatou E., Sklirou A., Kletsas D. (2015). Oxidative stress inhibits the proliferation, induces premature senescence and promotes a catabolic phenotype in human nucleus pulposus intervertebral disc cells. *European Cells & Materials*.

[63] Vergnes B., Gourbal B., Girard I., Sundar S., Drummelsmith J., Ouellette M. (2006). A proteomics screen implicates HSP83 and a small kinetoplastid calpain-related protein in drug resistance in *Leishmania donovani* clinical field isolates by modulating drug-induced programmed cell death. *Molecular & Cellular Proteomics*.

[64] Sereno D., Holzmuller P., Mangot I., Ouaissi A., Lemesre J., Cuny R. (2001). Antimonial-mediated DNA fragmentation in *Leishmania infantum* amastigotes. *Antimicrobial Agents and Chemotherapy*.

[65] Sudhandiran G., Shaha C. (2003). Antimonial-induced increase in intracellular Ca^2+^ through non-selective cation channels in the host and the parasite is responsible for apoptosis of intracellular *Leishmania donovani* amastigotes. *Journal of Biological Chemistry*.

[66] Paris C., Loiseau P. M., Bories C., Breard J. (2004). Miltefosine induces apoptosis-like death in *Leishmania donovani* promastigotes. *Antimicrobial Agents and Chemotherapy*.

[67] Verma N. K., Dey C. S. (2004). Possible mechanism of miltefosine-mediated death of *Leishmania donovani*. *Antimicrobial Agents and Chemotherapy*.

[68] Genois M. M., Plourde M., Ethier C. (2015). Roles of Rad51 paralogs for promoting homologous recombination in *Leishmania infantum*. *Nucleic Acids Research*.

[69] Moreira W., Leprohon P., Ouellette M. (2011). Tolerance to drug-induced cell death favours the acquisition of multidrug resistance in leishmania. *Cell Death & Disease*.

[70] Moreira W., Leblanc E., Ouellette M. (2009). The role of reduced pterins in resistance to reactive oxygen and nitrogen intermediates in the protozoan parasite *Leishmania*. *Free Radical Biology & Medicine*.

[71] Beverley S. M., Coderre J. A., Santi D. V., Schimke R. T. (1984). Unstable DNA amplifications in methotrexate resistant leishmania consist of extrachromosomal circles which relocalize during stabilization. *Cell*.

[72] Genois M. M., Paquet E. R., Laffitte M. C. N. (2014). DNA repair pathways in trypanosomatids: from DNA repair to drug resistance. *Microbiology and Molecular Biology Reviews*.

