# Supplementary material for: Oxidative Stress-Mediated Overexpression of Uracil DNA Glycosylase in Leishmania donovani Confers Tolerance against Antileishmanial Drugs
Source: Oxid Med Cell Longev. 2018 Feb 25;2018:4074357. doi: 10.1155/2018/4074357 (PMC5845521; doi:10.1155/2018/4074357)

Supplementary figures:


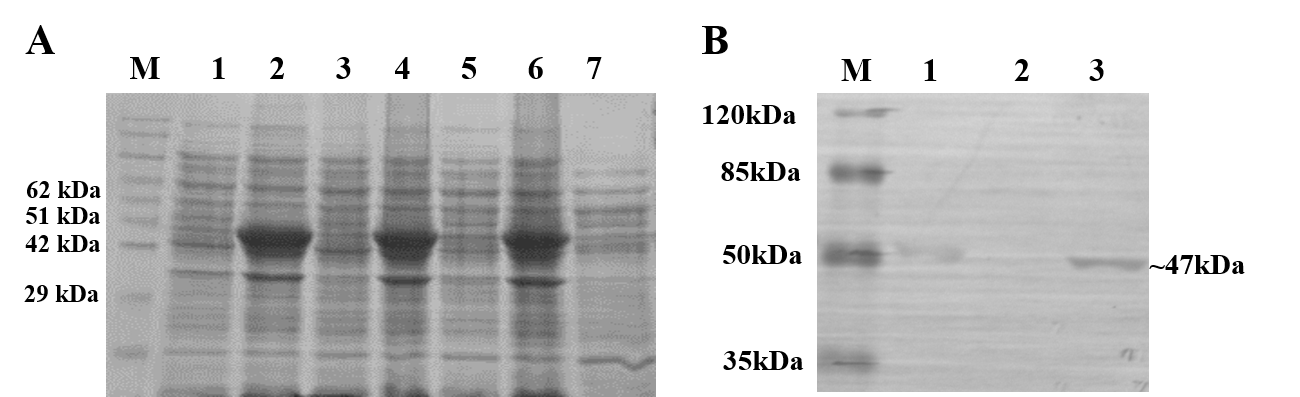


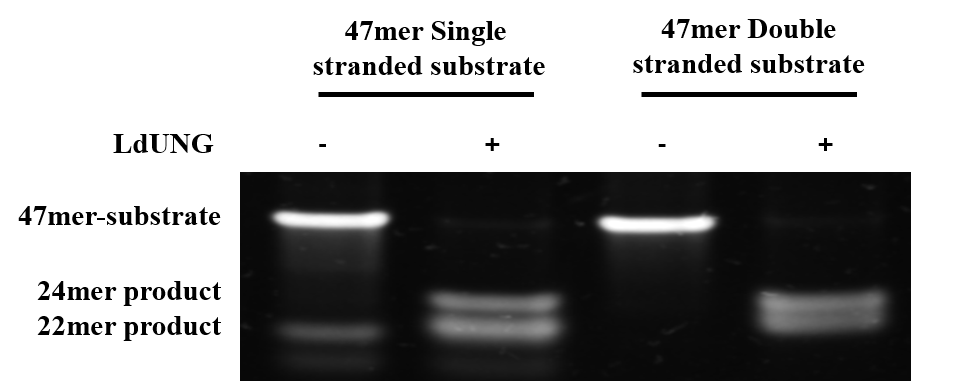
Fig. S1. Analysis of recombinant LdUNG expression by 12% SDS and western Blot. (A) SDSPAGE, M, unstained protein marker, Lane1, Uninduced supernatant; Lane 2, Induced pellet fraction (IPTG 0.2 mM); Lane 3, Induced supernatant fraction (IPTG 0.2 mM); Lane 4, Induced pellet fraction (IPTG 0.4 mM); Lane 5, Induced supernatant fraction (IPTG 0.4 mM); Lane 6, Induced pellet fraction (IPTG 0.6 mM); and Lane 7, Un-induced supernatant fraction. (B) Western Blot, Lane 3, purified LdUNG, Primary Ab: Anti His (dilution 1:12000), Secondary Ab: Anti Mouse conjugated with HRP (1:15000)

Fig. S2. Enzymatic activity of recombinant LdUNG on single and double stranded substrates.


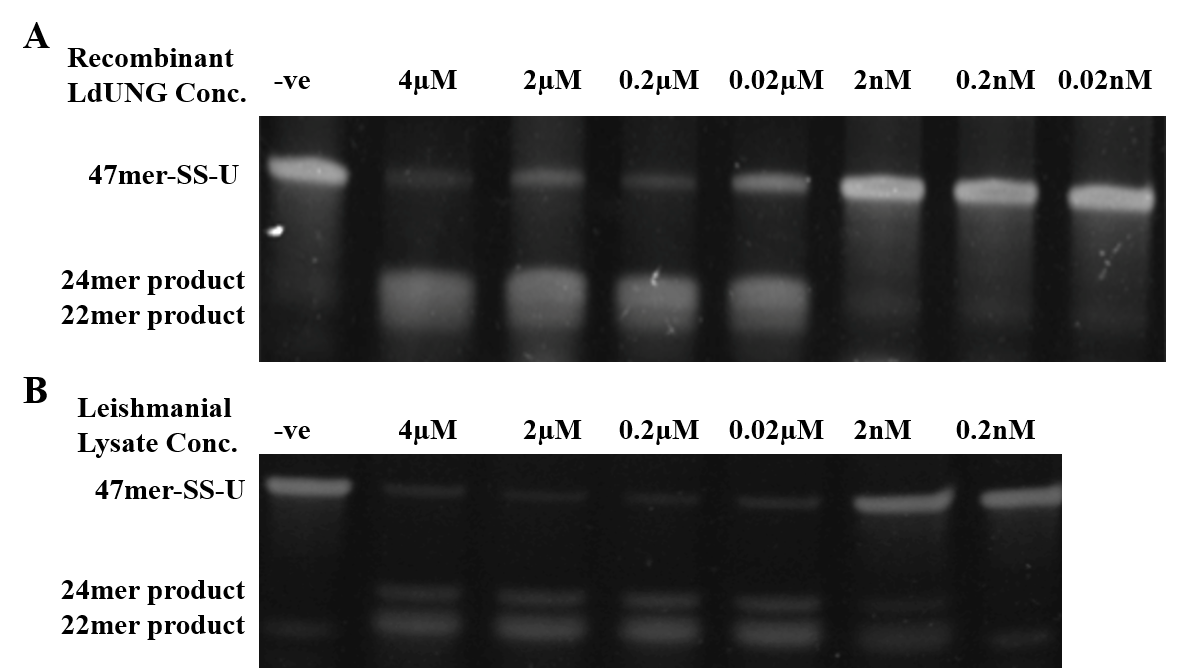


Fig. S3. Dose dependent enzymatic activity of Leishmania Uracil DNA glycosylase. The substrate (single stranded 47mer oligonucleotide having uracil at 23 position) was treated with recombinant LdUNG (A) and Leishmania lysate (B) as indicated.


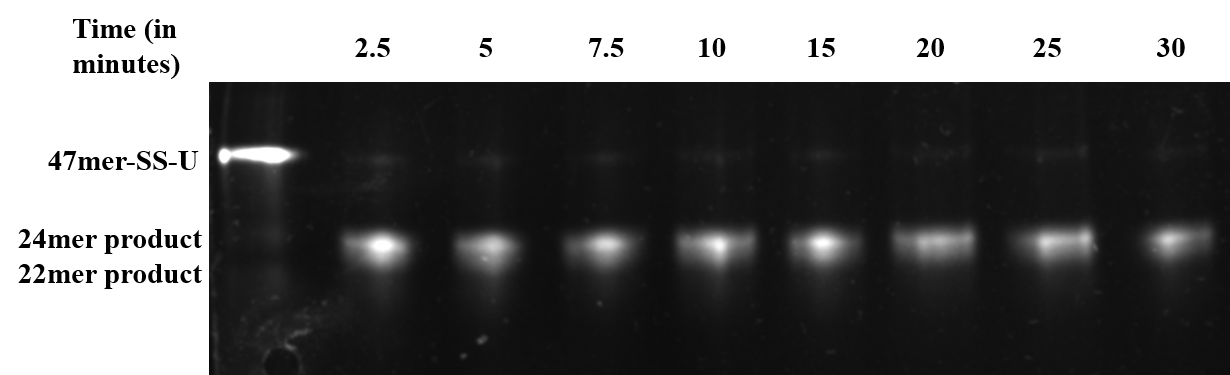


Fig. S4. Time dependent enzymatic activity of Leishmania Uracil DNA glycosylase. The substrate (single stranded 47mer oligonucleotide having uracil at 23 position) was treated with recombinant LdUNG for different incubation time as indicated.


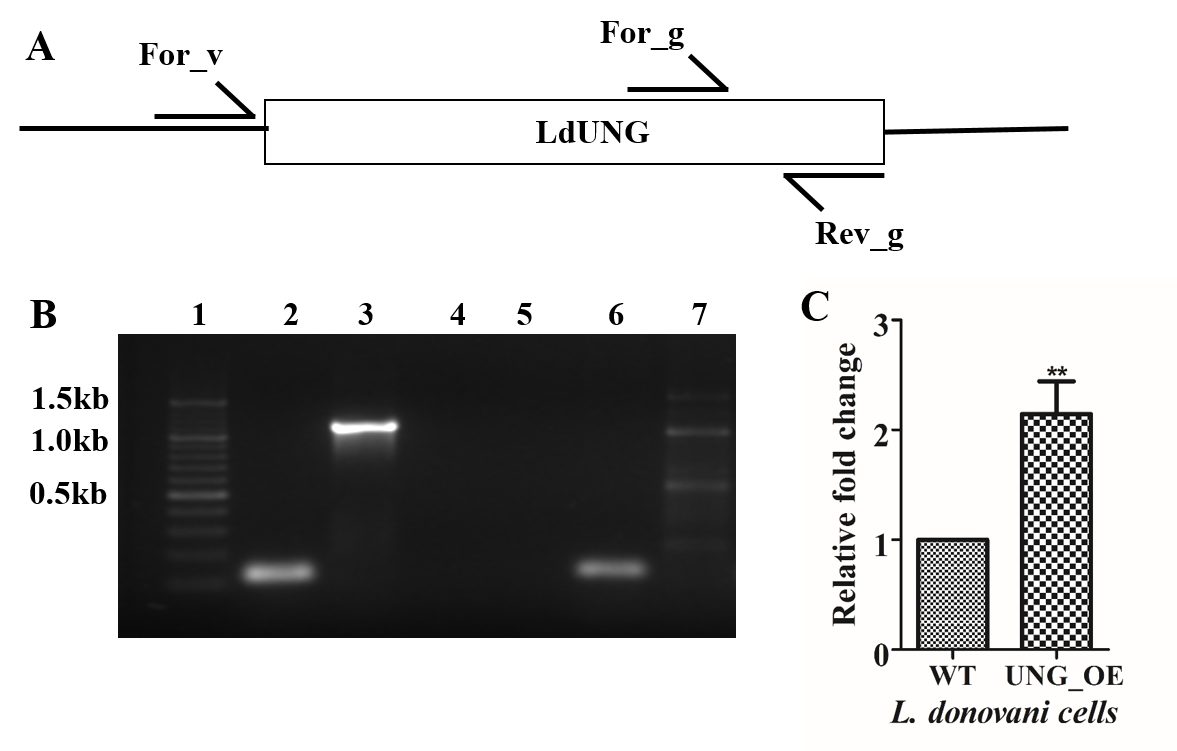


Fig. S5. (A) Schematic diagram showing primers used for confirmation of clone in transfected *L. donovani*. For_v indicate vector specific forward primer. For_g and Rev_g indicate gene specific forward and reverse primers, respectively. (B) Confirmation of LdUNG transfected *L. donovani* by PCR. Lane 1 indicates 100 bp DNA ladder. Lanes 2 and 3 represent PCR amplification using plasmid isolated from LdUNG transfectants. Lanes 4 and 5 contain PCR products amplified using vector pLPneo2 DNA. Lanes 6 and 7 contain PCR amplification using *L. donovani* genomic DNA as template. Lanes 3, 5 and 7 are amplification products with vector specific forward and gene specific reverse primers. Lanes 2, 4 and 6 represent PCR products using gene specific forward and reverse primers. (C) Relative abundance of UNG transcripts in WT and LdUNG transfectant determined by RT-PCR taking α-tubulin as internal control.


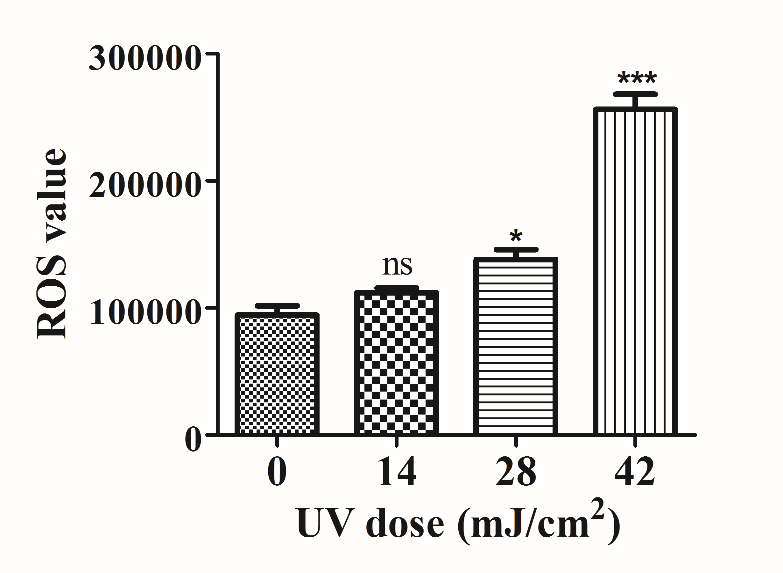


Fig. S6. Estimation of ROS in UV exposed *L. donovani* promastigotes. ROS was estimated in parasite cells after 30 min post UV (302 nm) treatment using CM-H2DCFDA as indicator.


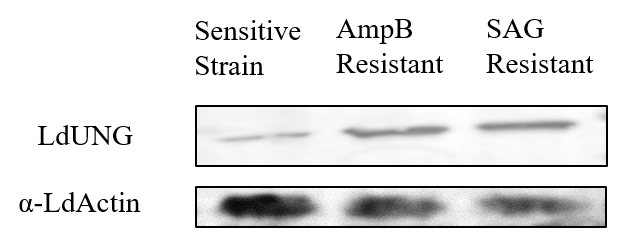


Fig. S7. Western Blot. Total cell lysates prepared from Sensitive, AmpB resistant and SAG resistant *L. donovani* strains were resolved on 10% SDS-PAGE and subjected to western blot analysis using LdUNG and α-LdActin antibodies, respectively. Primary Ab: Anti LdUNG (dilution 1:5000), Secondary Ab: Anti Rabbit conjugated with HRP (1:15000)

Table S1. Sequences of oligonucleotides used as substrates in UNG activity assay.


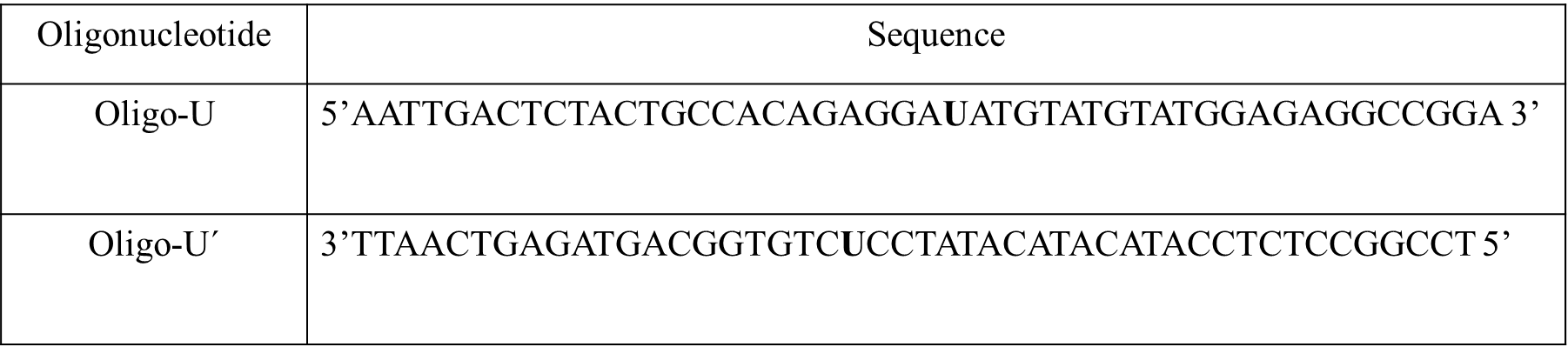

Supplement: Supplementary Materials — Figure S1: analysis of recombinant LdUNG expression by 12% SDS and Western blot. (A) SDS-PAGE: M, unstained protein marker; lane 1, uninduced supernatant; lane 2, induced pellet fraction (IPTG 0.2 mM); lane 3, induced supernatant fraction (IPTG 0.2 mM); lane 4, induced pellet fraction (IPTG 0.4 mM); lane 5, induced supernatant fraction (IPTG 0.4 mM); lane 6, induced pellet fraction (IPTG 0.6 mM); and lane 7, uninduced supernatant fraction. (B) Western blot: lane 3, purified LdUNG; primary Ab, anti-His (dilution 1 : 12000); secondary Ab, anti-mouse conjugated with HRP (1 : 15000). Figure S2: enzymatic activity of recombinant LdUNG on single- and double-stranded substrates. Figure S3: dose-dependent enzymatic activity of leishmania uracil DNA glycosylase. The substrate (single-stranded 47-mer oligonucleotide having uracil at 23 position) was treated with recombinant LdUNG (A) and leishmania lysate (B) as indicated. Figure S4: time-dependent enzymatic activity of leishmania uracil DNA glycosylase. The substrate (single-stranded 47-mer oligonucleotide having uracil at the 23rd position) was treated with recombinant LdUNG for different incubation time as indicated. Figure S5: (A) schematic diagram showing primers used for confirmation of clone in transfected L. donovani. For_v indicates vector-specific forward primer. For_g and Rev_g indicate gene-specific forward and reverse primers, respectively. (B) Confirmation of LdUNG-transfected L. donovani by PCR. Lane 1 indicates 100 bp DNA ladder. Lanes 2 and 3 represent PCR amplification using plasmid isolated from LdUNG transfectants. Lanes 4 and 5 contain PCR products amplified using vector pLPneo2 DNA. Lanes 6 and 7 contain PCR amplification using L. donovani-genomic DNA as template. Lanes 3, 5, and 7 are amplification products with vector-specific forward and gene-specific reverse primers. Lanes 2, 4, and 6 represent PCR products using gene-specific forward and reverse primers. (C) Relative abundance of UNG transcripts in [file 4074357.f1.docx]
